# Illumina MiSeq Phylogenetic Amplicon Sequencing Shows a Large Reduction of an Uncharacterised Succinivibrionaceae and an Increase of the *Methanobrevibacter gottschalkii* Clade in Feed Restricted Cattle

**DOI:** 10.1371/journal.pone.0133234

**Published:** 2015-07-30

**Authors:** Matthew Sean McCabe, Paul Cormican, Kate Keogh, Aaron O’Connor, Eoin O’Hara, Rafael Alejandro Palladino, David Anthony Kenny, Sinéad Mary Waters

**Affiliations:** 1 Animal and Bioscience Research Department, Animal and Grassland Research and Innovation Centre, Teagasc, Grange, Dunsany, County Meath, Ireland; 2 UCD School of Agriculture, Food Science and Veterinary Medicine, College of Life Sciences, University College Dublin, Belfield, Dublin, Ireland; 3 Department of Animal Production, University of Buenos Aires, Buenos Aires City, Argentina; University of Illinois, UNITED STATES

## Abstract

Periodic feed restriction is used in cattle production to reduce feed costs. When normal feed levels are resumed, cattle catch up to a normal weight by an acceleration of normal growth rate, known as compensatory growth, which is not yet fully understood. Illumina Miseq Phylogenetic marker amplicon sequencing of DNA extracted from rumen contents of 55 bulls showed that restriction of feed (70% concentrate, 30% grass silage) for 125 days, to levels that caused a 60% reduction of growth rate, resulted in a large increase of relative abundance of *Methanobrevibacter gottschalkii* clade (designated as OTU-M7), and a large reduction of an uncharacterised Succinivibrionaceae species (designated as OTU-S3004). There was a strong negative Spearman correlation (*ρ* = -0.72, P = <1x10^-20^) between relative abundances of OTU-3004 and OTU-M7 in the liquid rumen fraction. There was also a significant increase in acetate:propionate ratio (A:P) in feed restricted animals that showed a negative Spearman correlation (*ρ* = -0.69, P = <1x10^-20^) with the relative abundance of OTU-S3004 in the rumen liquid fraction but not the solid fraction, and a strong positive Spearman correlation with OTU-M7 in the rumen liquid (*ρ* = 0.74, P = <1x10^-20^) and solid (*ρ* = 0.69, P = <1x10^-20^) fractions. Reduced A:P ratios in the rumen are associated with increased feed efficiency and reduced production of methane which has a global warming potential (GWP 100 years) of 28. Succinivibrionaceae growth in the rumen was previously suggested to reduce methane emissions as some members of this family utilise hydrogen, which is also utilised by methanogens for methanogenesis, to generate succinate which is converted to propionate. Relative abundance of OTU-S3004 showed a positive Spearman correlation with propionate (*ρ* = 0.41, P = <0.01) but not acetate in the liquid rumen fraction.

## Introduction

Cattle and other domestic ruminants, such as sheep and goats, are economically important because of their ability to convert low-quality forages into high quality, high protein products (milk and meat) suitable for human consumption [1]. This ability is largely due to the microbial community in the rumen which is highly adapted to the breakdown and fermentation of lignocellulose, the most abundant carbon polymer on earth [2]. The rumen therefore plays a central role in unlocking this vast energy store which is largely inaccessible to the human digestive system [3]. In addition to the structural carbohydrates (e.g. cellulose and hemicellulose), which are a large component of low-quality forages (e.g. straw and grass), other types of cattle feed such as grains and legumes also comprise high quantities of non-structural carbohydrates (e.g. starch), pectin, proteins and lipids [4].

The most important by-products of rumen microbial metabolism are the volatile fatty acids (VFAs), mostly acetate, propionate and butyrate. VFAs are absorbed across the rumen wall, into the bloodstream and provide cattle with up to 70% of their energy [5]. However, the amount of energy supplied from feed to cattle by rumen fermentation is highly variable and dependent on the feeding strategy. Certain feeding conditions (e.g. low intake of low digestibility diets) lead to increased losses of energy to methane production [6]. Methane is generated by several species of ‘methanogenic’ rumen archaea, which use rumen fermentation products (e.g. hydrogen and carbon dioxide, formate, or methyl compounds) as substrates for growth [7]. Acetate, while used as a growth substrate by methanogens in other environments, is not metabolised to methane to any significant extent in the rumen. It has been known for many years that increasing intake of highly digestible feed leads to lower methane loss per kg of feed [6]. This causes a change in the ratio of the rumen microbe-derived VFAs with a reduction of acetate and an increase of propionate. Decreased acetate:propionate ratio (A:P) has been shown to be associated with decreased methane emissions [8]. Unlike acetate, propionate metabolism in the rumen does not result in the generation of methane and so is energetically more efficient than other rumen VFAs [9]. Addition of monensin to cattle feed to increase the concentration of propionate in the rumen without affecting overall VFAs was first reported in the 1970s [10] and was also widely adopted to reduce methane and increase feed efficiency in cattle production, although its use in cattle was phased out in the EU in 2006. However, although many of the effects of feed type and intake on rumen VFAs and consequent methane production have been known for more than 50 years, the activities of the rumen microbiota that are associated with these changes are still not fully understood.

A comprehensive and detailed understanding of the rumen microbiome remains far from complete largely because it is currently not possible to culture the majority of its thousands of constituent microbial species [11], most of which represent <1% of total microbial species in a single rumen. However, the recent rapid technical advances in length and number of high quality DNA sequencing reads using next generation sequencing (NGS) means that culture-free approaches, such as metagenomics, metatranscriptomics and phylogenetic gene marker sequencing, now allow us to view the entire rumen microbiota at far greater depth and resolution than was previously possible [12].

A strategy commonly used in the cattle industry to reduce feed costs is to periodically restrict the amount of feed given to the animal, particularly during the winter when feed is expensive. When cattle are returned to a normal diet after a period of feed restriction, their growth rate significantly increases compared to animals which have not been subjected to feed restriction. This increased growth rate is termed ‘compensatory growth’ and allows animals to achieve a normal slaughter weight within a normal growth period but with lower feed costs [13]. In a compensatory growth model that we conducted on Holstein-Friesian bulls [14] we found that bulls that were subjected to 125 days of feed restriction showed a large increase in the rumen A:P ratio compared to bulls which had been continuously fed an *ad libitum* diet. At 55 days post-restriction these A:P ratios had returned to normal levels. In the work we present here, the aims were to use 16S Illumina amplicon sequencing to (i) identify the changes in the rumen microbiota associated with increased A:P ratios in feed restricted animals, (ii) determine whether feed restriction caused long lasting effects on the composition of the rumen microbiota after re-feeding, and (iii) assess the extent to which the rumen microbiota might contribute to compensatory growth.

The main findings from this were that phylogenetic amplicon sequencing of DNA extracted from rumen contents (liquid and solid) of feed restricted animals showed a large increase in species diversity, a large decrease in relative abundance of an abundant operational taxonomic unit (OTU) that was identified as an uncultured member of Succinivibrionoaceae, and a significant increase in only one of the two most abundant methanogen OTUs that were detected. The methanogen OTU that increased in feed restricted animals is a member of the *Methanobrevibacter gottschalkii* clade. Fifty five days after feed restricted animals were returned to a normal diet, although they showed accelerated growth, their rumen microbiomes were not different to control animals that had not been subjected to feed restriction. This showed that the disruption caused by feed restriction did not have long term effects on the rumen microbiome composition. It also showed that the rumen prokaryotic microbiome was not associated with compensatory growth 55 days post-restriction.

## Methods

### Animal model

**Fig 1:**
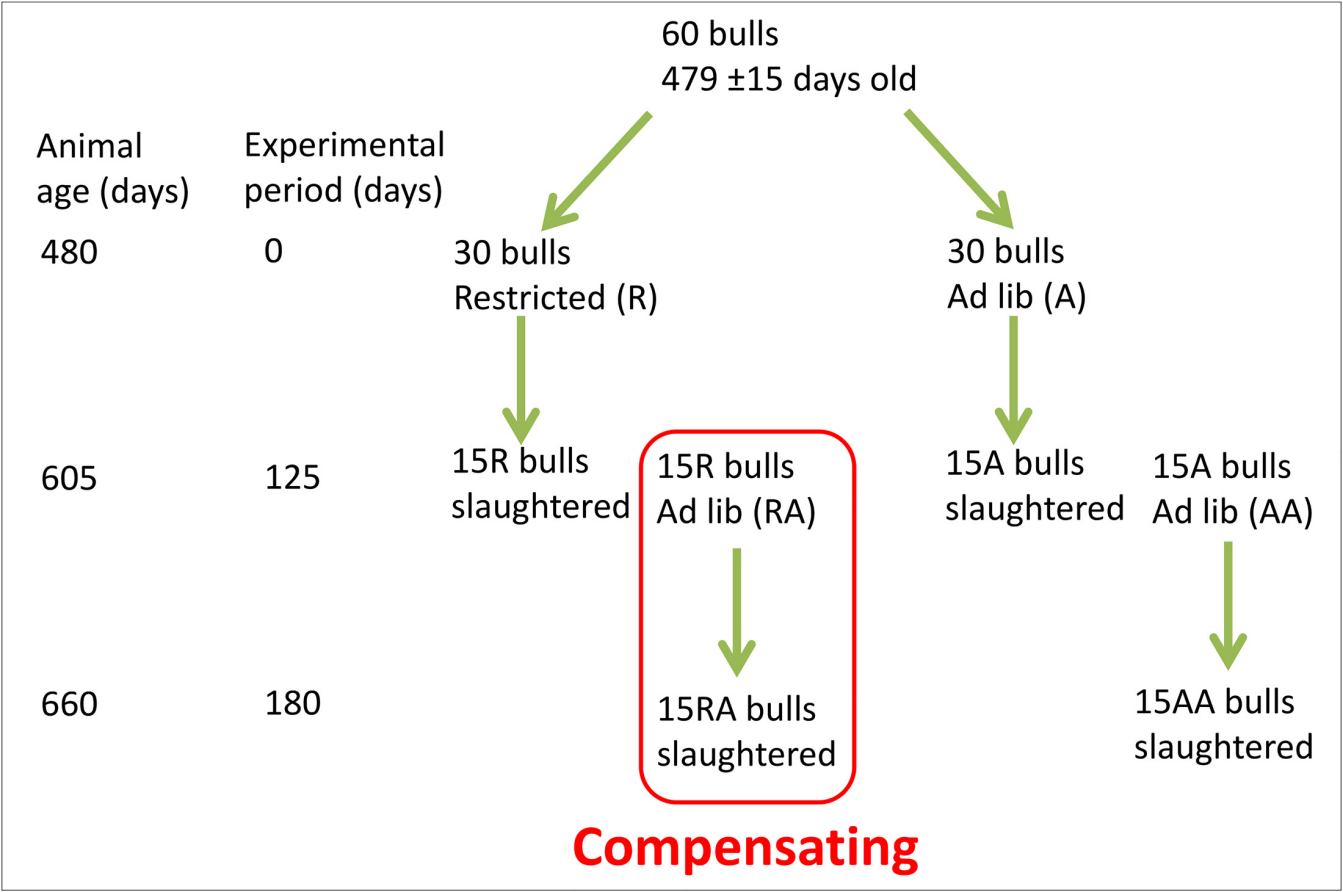
Scheme of compensatory growth animal model.

All procedures involving animals were approved by the University College Dublin, Animal Research Ethics Committee and licensed by the Irish Department of Health and Children in accordance with the European Community Directive 86/609/EC. Animals were slaughtered using standard procedures (captive bolt stunning and exsanguination) at a licensed abattoir. An outline of the model is shown in Fig 1. A group of 60 Holstein-Friesian bulls (479 ±15 days old) was divided into two groups of 30 animals. One group was subjected to an *ad libitum* diet (group A) and the other group was subjected to a restricted diet (group R). Both groups A and R received a 70% concentrate, and 30% grass silage diet. The concentrate formulation was: rolled barley 72.5%, soya 22.5%, molasses 3%, calf mineral 2%. Group R animals were fed to grow at 0.6 kg/day for 125 days and animals on the *ad libitum* diet were predicted to grow in excess of 1.5 kg per day. For this, diets were calculated daily for each animal and fed individually, using Calan gates (American Calan Inc., Northwood, NH) with the proportion of feed required estimated based on each animal’s own live weight and expected rate of gain. At 125 days, 15 animals from each group (R and A) were slaughtered (September, 2011) and rumen contents were collected. The remaining 15 animals from both group R and group A were then offered an *ad libitum* diet for a further 55 days, slaughtered (November, 2011) and their rumen contents collected. These animals were designated as RA and AA. In summary, there were four groups of bulls R, A, RA and AA. All bulls received the same high digestibility diet throughout the experiment except that the diet was restricted in one group. Group R was on the restricted diet for 125 days, group A was on an *ad libitum* diet for 125 days, group RA was on a restricted diet for 125 days then an *ad libitum* diet for 55 days (compensatory growth period), group AA was on an *ad libitum* diet for 180 days. One group R animal, two group A animals and two group AA animals were removed from the study due to illness, leaving 14 R, 13 A, 15 RA, 13 AA and animals.

### VFA analysis

Rumen digesta were sampled from five different points within the rumen of each bull at slaughter, including the dorsal and ventral sacs. The digesta samples were filtered through cheese cloth and the liquid was collected. 0.5 mL of 9 M sulphuric acid were added to 20 mL of the filtered rumen liquid, mixed and stored at -20°C for subsequent analysis of VFAs. The concentrations of VFAs were measured using an automated gas chromatograph (Shimadzu Gas Chromatography GC-8A, Shimadzu Corporation, Kyoto, Japan) [15].

### DNA extraction

Rumen contents, liquid and solid, were collected immediately after slaughter from five different points within the rumen of each bull, including the dorsal and ventral sacs. Contents were squeezed through cheese cloth to separate the rumen liquid and solid fractions. Solid and liquid fractions were immediately frozen in liquid nitrogen, transported on dry ice then stored at -80°C. Approximately 20 g of frozen rumen solid or liquid sample from each of the animals was considered as representative. Each sample (approximately 20 g) was homogenised to a fine frozen powder under liquid nitrogen in a pestle and mortar. The frozen powder was stored at -80°C. Approximately 250 mg and 600 mg of the homogenized frozen powder was used for DNA extraction from the solid and liquid fractions respectively. DNA was extracted using the repeated bead beating and column purification method [16]. DNA quality was assessed on an agarose gel. DNA was quantified by heating at 52°C for 2 min, gently mixing, then taking three consecutive readings on the Nanodrop 1000 spectrophotometer. Nanodrop 1000 readings were within 5% of each other.

### Library preparation and sequencing

We generated 110 amplicon libraries by PCR amplification of the hypervariable (V4) region of the 16S rRNA gene in bacteria and archaea from 20 ng of rumen DNA that had been extracted from either the solid (S) or liquid (L) fractions of rumen contents from 55 individual bulls from the four treatment groups R, A, RA and AA. PCR was performed with barcoded 16S Illumina adapter fusion 515F/806R rcbc primers, which contain 12 bp barcodes [17, 18], using Q5 Hot Start High-Fidelity DNA Polymerase (New England Biolabs Inc.). Cycle conditions were 98°C (30 s), then 30 cycles of 98°C (10 s), 72°C (30 s), (annealing and polymerisation steps were both performed at the same temperature) then a final extension of 72°C (2 min). Libraries were purified using the QIAquick PCR Purification Kit (Qiagen, UK) and then measured for purity and quantity on the Nanodrop 1000. The 110 libraries were designated according to rumen fraction and treatment group as follows: SR (n = 14), SA (n = 13), SRA (n = 15), SAA (n = 13), LR (n = 14), LA (n = 13), LRA (n = 15) and LAA (n = 13). The barcoded amplicon libraries were then combined into 4 pools (SR+SA, SRA+SAA, LR+LA and LRA+LAA) in equal concentrations and each pool was gel purified to remove primer dimers using the QIAquick Gel Extraction Kit (Qiagen). An extra purification with the QIAquick purification kit was used to remove residual agarose. The pools of gel-purified libraries were then measured for purity and quantity on the Nanodrop 1000 and further quantified using the KAPA SYBR FAST Universal qPCR kit with Illumina Primer Premix (Kapabiosystems). The library pools were then diluted and denatured according to the Illumina MiSeq library preparation guide. 6 pM amplicon library was spiked with 30% denatured and diluted PhiX Illumina control library version 3 (12.5 pM). Four sequencing runs (one library pool per run) were conducted on the Illumina MiSeq using 300 cycle MiSeq reagent kits (version 2). Approximately 240,000–260,000 merged reads were generated for each of the 110 amplicon libraries.

### Sequence quality control and pre-processing

Demultiplexing of sequence reads was carried out using an in-house perl script. Raw sequence read pairs were quality trimmed and filtered to remove sequencing adaptor contamination using Trim Galore [19]. Remaining read pairs were each merged into a single contiguous sequence. Size selection of 254 bp ±20 bp sequences was carried out using an in-house perl script. Reads from all samples were subsequently combined into a single dataset for processing with QIIME [20].

### Amplicon sequencing and data analysis

A combination of *de novo* and reference based OTU identification was carried out using the open reference calling method implemented within QIIME. A default similarity level of 97% was used to cluster sequences into individual OTUs and a single representative sequence from each clustered OTU was used to align to the Greengenes database (version: gg_13_5) [21]. Taxonomic classification for each OTU was determined with RDP Classifier [22] using a minimum confidence cut off of 0.8. OTUs with fewer than 100 sequences across all samples were excluded from further analysis. Rarefaction analysis confirmed that sequencing of each sample was conducted to sufficient depth. OTUs and their relative abundances in each sample are given in S1 Table.

### Statistics

Significant differences of relative abundance of all OTUs between pairs of treatment groups (LR vs. LA, SR vs. SA, LRA vs. LAA and SRA vs. SAA) were calculated in Python [23] using the Wilcoxon rank sum test with correction for multiple testing using the Benjamini Hochberg (BH) method [24]. A false discovery rate (FDR) of <0.05 was considered to be significant. Spearman correlation analysis was conducted using GenEx Pro multid software [25]. Box and whisker plots and the principal components analysis (PCA) loading plot were prepared in Minitab [26]. Multiple comparisons of relative abundance of single OTUs in all treatment groups were conducted in R [27] using the pairwise Wilcoxon test (R function = pairwise.wilcox.test) with BH correction for multiple testing. Significant differences in animal weights were calculated using a t-test (two-tailed, two-sample unequal variance) in Microsoft Excel 2010.

## Results and Discussion

### Animal model

Average daily feed intake (kg) for each animal group was R = 5.44 ±0.17, A = 12.80 ±0.43, RA = 11.8 ±0.97 and AA = 12.22 ±0.98. The average daily increase in weight (kg) for each group was R = 0.62 ±0.07, A = 1.93 ±0.15, RA = 2.5 ±0.52 and AA = 1.4 ±0.30. The average live slaughter weight (kg) of group R animals was significantly less (P = 1.67 x10^-10^) than group A animals (R = 466.1 ±44.6, A = 631.8 ±41.6) and the live weight of group RA animals was significantly less (P = 1.58 x 10^−5^) than group AA animals (RA = 575.5 ±36.9, AA = 667.1 ±56.0) animals. This confirmed that feed restriction caused restricted growth rate in group R bulls as expected and that group RA bulls were still undergoing compensatory growth when rumen samples were collected at slaughter.

### Differences between restricted and ad libitum diet rumen prokaryotes

**Fig 2:**
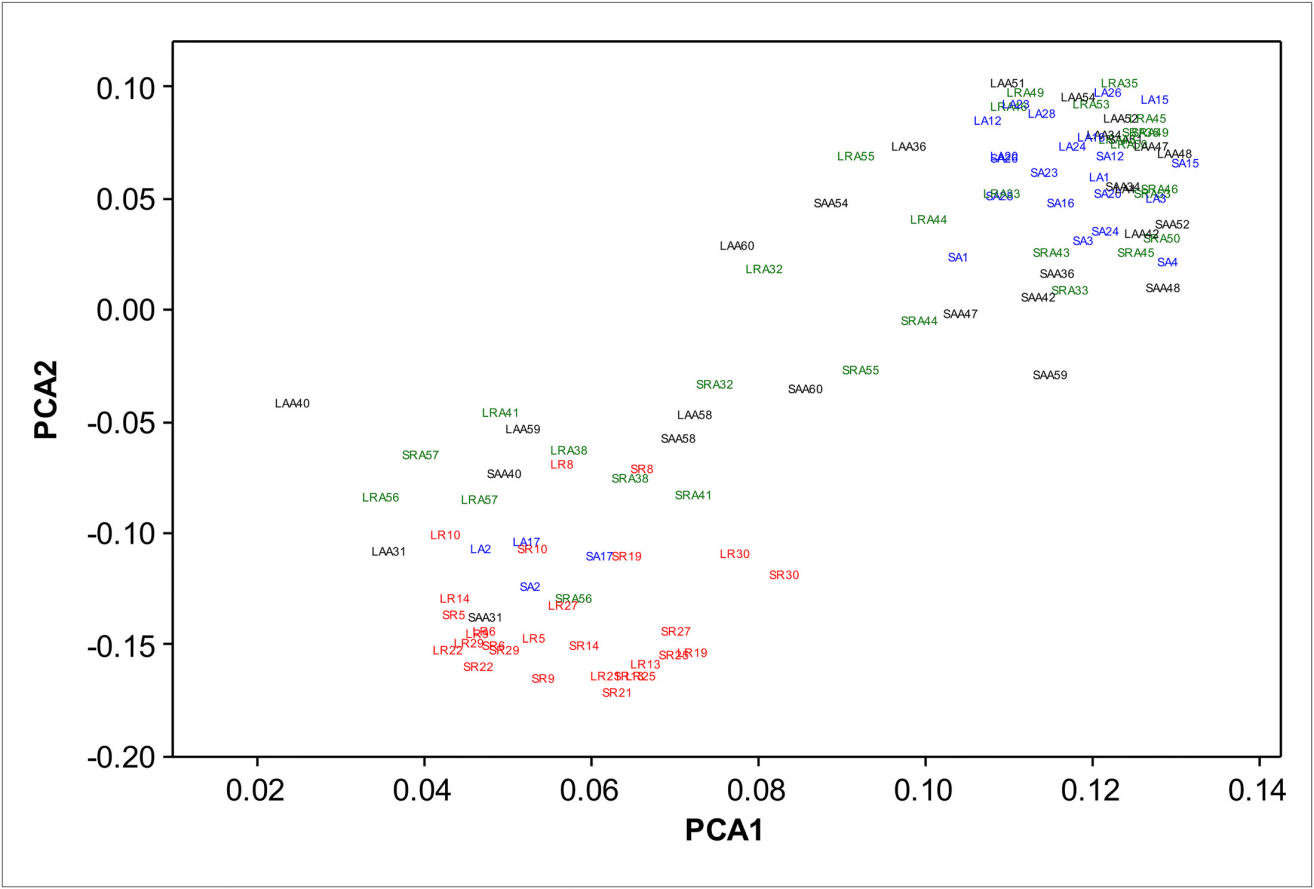
Principal components analysis (PCA) of relative abundances. PCA was used to compare variation of relative OTU abundances within and between liquid (L) and solid (S) rumen samples from groups R, A, RA and AA. Variation in individual samples is shown. Red = group R (LR and SR), blue = group A (LA and SA), green = group RA (LRA and SRA) and black = group AA (LAA and SAA). Individual animals are indicated by numbers (e.g. LRA55 and SRA55 are animal number 55, liquid and solid respectively, treatment group RA).

Principal components analysis (PCA) of relative abundances of OTUs from groups R, A, RA and AA OTUs showed that, apart from 2 animals (A2 and A17), group R separated from group A in the first and second principal components for both liquid and solid fractions (Fig 2). We are uncertain why rumen microbe profiles of A2 and A17 were more similar to group R than group A as their intakes and weight increase were not unusually low for group A. The slaughter weights and carcass weights of animals A2 and A17 were the lowest and third lowest respectively in group A but still higher than the group R average. Animals A1, A4 and A15 also had low group A slaughter and carcass weights and yet their microbiome profiles were very different to those of group R. Comparison of relative abundances of OTUs detected in group R with those in group A showed that 58% (solid fraction) and 55% (liquid fraction) of the OTUs were significantly different (FDR<0.05) between these two groups (S1 Table). So it is clear that feed restriction caused large changes in the composition of the rumen microbiome.

**Fig 3:**
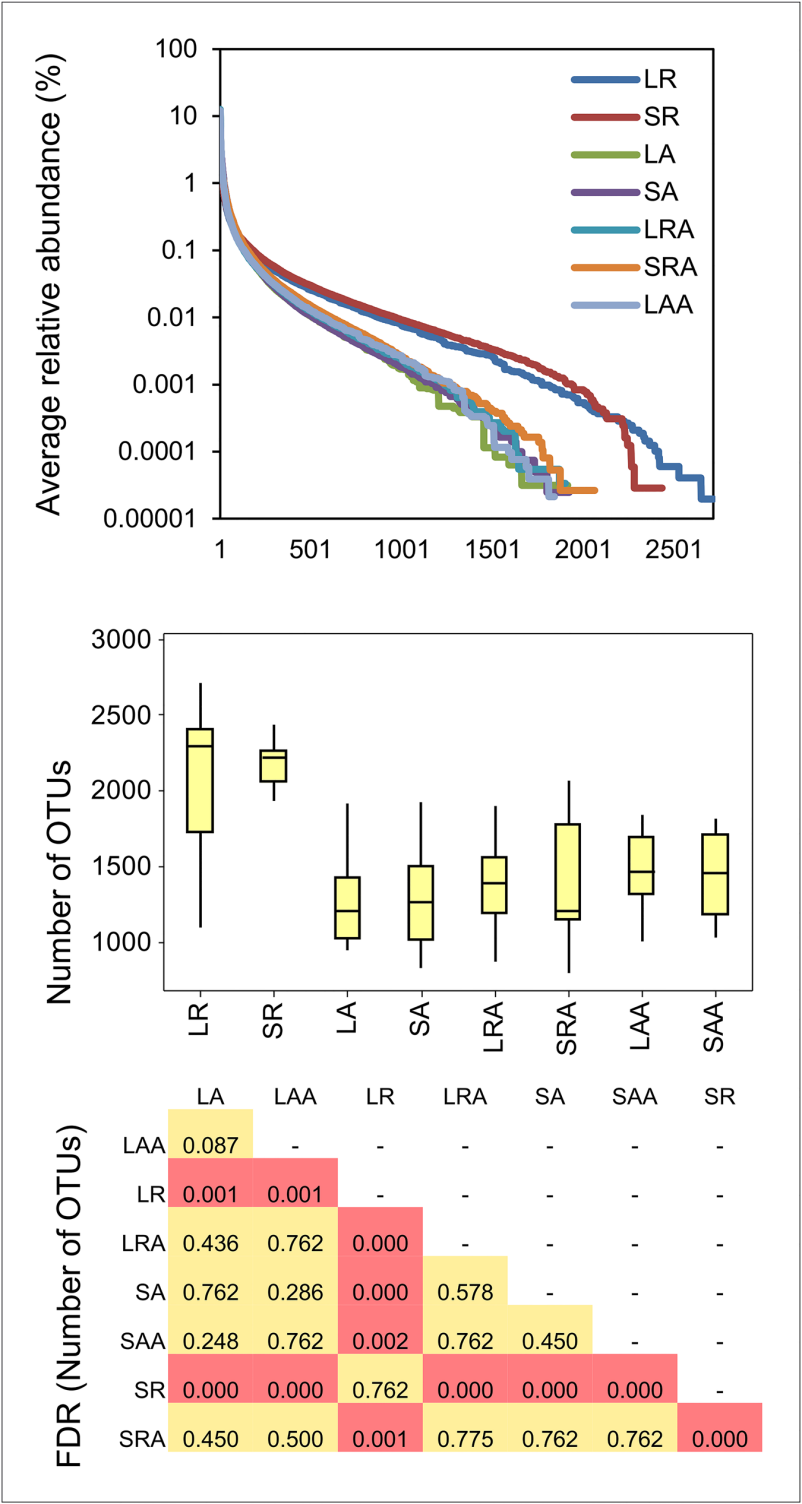
Difference between diversity of OTUs in rumens of restricted and *ad libitum* treatment groups LR = Liquid Restricted (n = 14), SR = Solid Restricted (n = 14), LA = Liquid *Ad lib* (n = 13), SA = Solid *Ad lib* (n = 13). Line plot (top panel) shows an increase in group R relative to the other groups, in the average number of OTUs for which the relative abundance <0.1%. Box and whisker plot shows median, quartiles and maximum and minimum values of the sums of the number of OTUs identified within in each sample. P-values are BH-corrected and derived from Wilcoxon pairwise comparisons of number of OTUs between different treatment groups.

The diversity of prokaryotic OTUs was also greatly increased in group R compared to groups A, RA and AA (Fig 3). This increased diversity was probably due to the slower passage rate in group R which would have allowed the slower growing microbes to become established. There have been numerous studies spanning several decades on the effects of feed restriction on rumen fermentation activity and these generally show that as long as maintenance requirements are met, as was the case in group R, there is a negative relationship between level of feed intake and digestability. This is mostly attributed to slower passage rate of feed and increased particle retention time in the rumen when intake decreases [28, 29] but could also be due to increased microbial diversity. If the increased microbial diversity in the rumen persisted for the early part of the post-restriction period, this may have contributed to improved fermentation of the *ad libitum* diet in the group RA (compensating) animals compared to the group AA animals. Unfortunately we only took samples for DNA extraction and VFA analysis at 55 days post-restriction so it was not possible to determine how many days post-restriction that it took for the rumen microbiota to revert to the *ad libitum* state in group RA animals.

### Comparison of rumen prokaryotes between *ad libitum* diet groups

In contrast, PCA showed no obvious separation of OTU relative abundances for groups RA and AA and there was more variation between animals within groups RA and AA than within groups R and A (Fig 2). In addition, there were no significant differences (FDR<0.05) when the relative abundance of each OTU was compared between group RA and AA (S1 Table). This suggests that 55 days after returning to the *ad libitum* diet, the rumen microbiota of most of the restricted animals had essentially recovered from the effects of long-term feed restriction. However, the rumen microbiota profiles for several of the group AA and group RA animals appeared to be more similar to the restricted group than the *ad libitum* groups. We are not certain of the cause of this as animal weights and intake of these animals was not different to the other *ad libitum* group animals. In addition to the similarity of microbiota between group RA and AA, we found that the size and weight of the rumen and liver, and global gene expression in liver and rumen wall (determined by RNAseq analysis), while very different between group R and group A, were not different between group RA and group AA [14, 30, 31]. This is despite the fact that group RA animals had an 80% higher growth rate than group AA animals at the 55 day sampling time point. So it is likely that the major physiological changes, possibly including the rumen, that contributed to compensatory growth in the post-restriction period occurred earlier than the 55 day sampling time point, and that the increased growth rate in group RA at 55 days post-restriction was caused by residual effects of these changes that were occurring elsewhere in the animal. We also conducted RNAseq analysis on muscle biopsies that were collected at 15 days post-restriction and found that transcript profiles were very different between group RA and group AA showing that major changes were occurring in muscle in compensating animals at around two weeks post-restriction [32]. So far we have not conducted RNAseq analysis on muscle samples taken 55 days post-restriction. Meiske et al. (1958) reported that a 72 hour starvation period led to a decline in numbers of bacteria and cellulolytic activity in rumen fluid taken from four year old fistulated steers on a high forage diet [33]. These returned to normal just 4 days after refeeding. Warner (1962) found that starvation led to disappearance of several species of protozoa and bacteria in sheep rumen and that these species took between 3 to 55 days reappear [34]. Zhang et al. (2013) reported that rumen propionate levels decreased in cannulated Angus x Hereford heifers that had their feed restricted for 5 days to 25% of the normal levels. In their model, at 7 days post-refeeding, propionate levels were increased relative to 75% restricted animals, then were at near normal levels at two and three weeks post-refeeding [35]. In our model, similar changes in the rumen that may have contributed to compensatory growth could have occurred in the first one or two weeks post-restriction and, even though animals were exhibiting accelerated growth, our 55 day post-restriction sampling time point was too late to detect these changes.

### Reduction of abundant putative Succinivibrionaceae OTU in feed restricted animals

**Fig 4:**
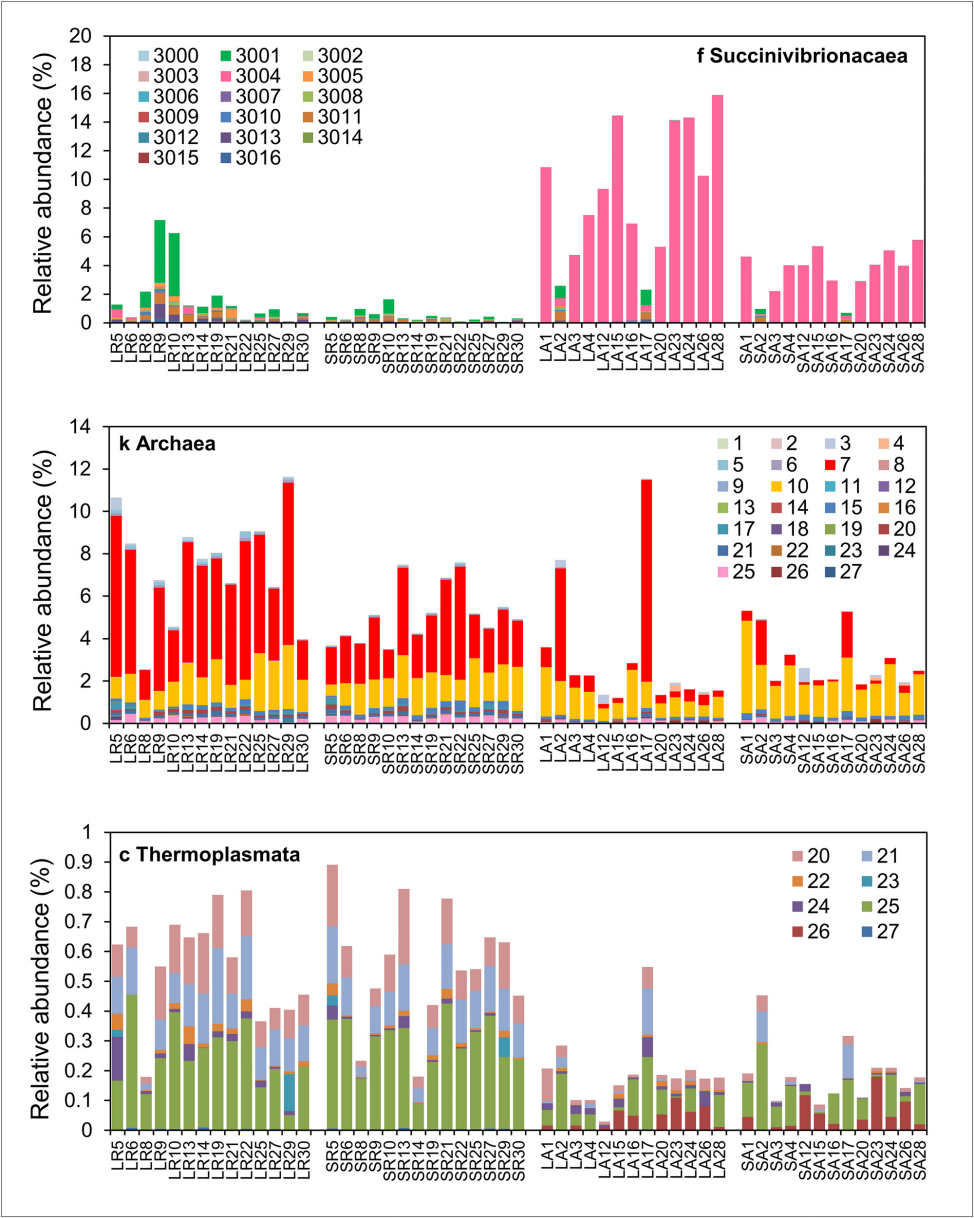
Relative abundance of individual methanogen and Succinivibrionaceae OTUs in group R and group A. Relative abundance of OTUs identified as family Succinivibrionaceae, kingdom Archaea or class Thermoplasmata (recently reclassified as members of the order Methanomassiliicoccales) in individual liquid (L) and solid (S) rumen samples from groups R and A are shown. Different colours represent different OTU numbers. Taxonomic assignments for all OTU numbers are given in S1 Table.

**Fig 5:**
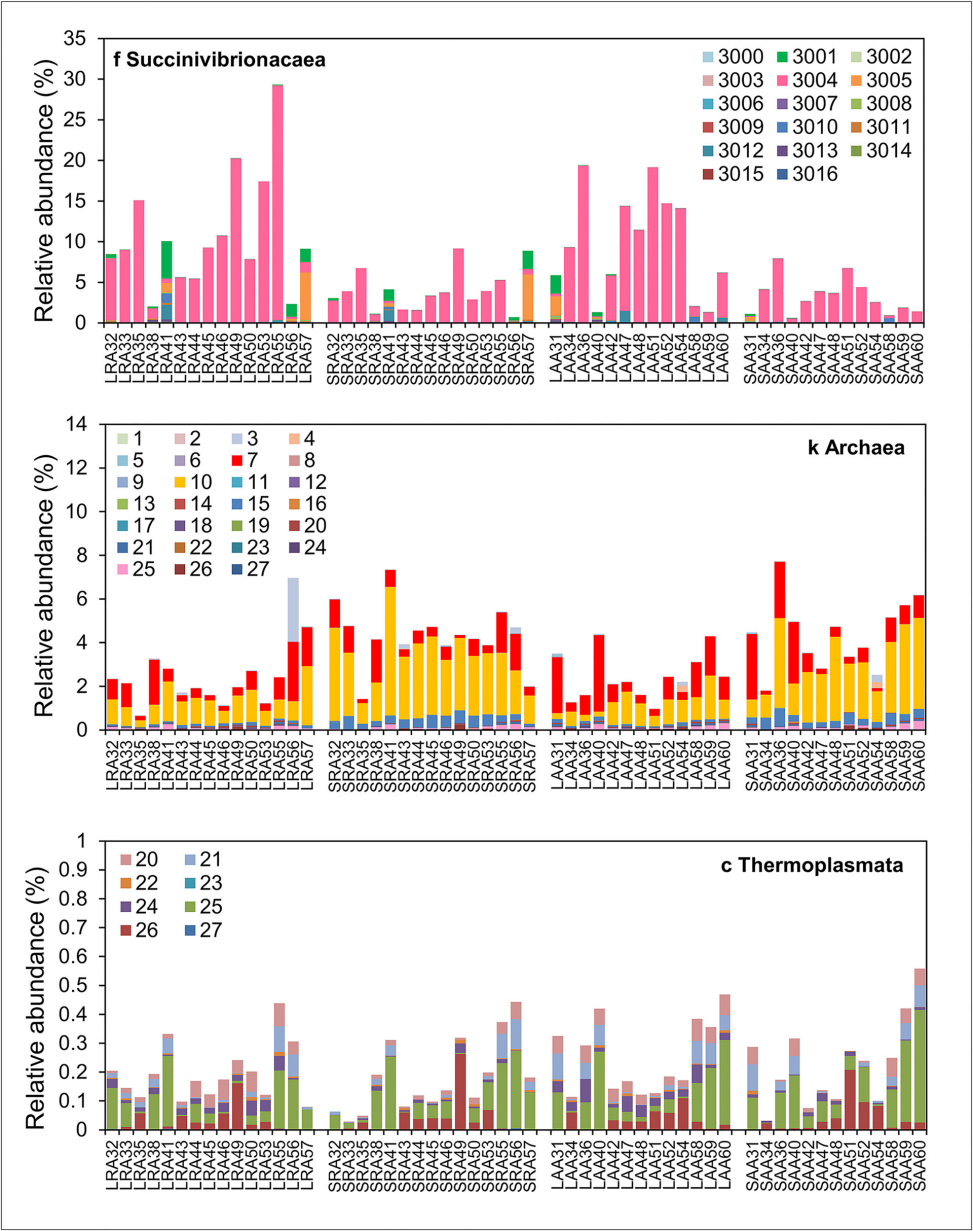
Relative abundance of individual methanogen and Succinivibrionaceae OTUs in group RA and AA. Relative abundance of OTUs identified as family Succinivibrionaceae, kingdom Archaea or class Thermoplasmata (recently reclassified as members of the order Methanomassiliicoccales) in individual liquid (L) and solid (S) rumen samples from groups RA and AA are shown. Different colours represent different OTU numbers. Taxonomic assignments for all OTU numbers are given in S1 Table.

**Fig 6:**
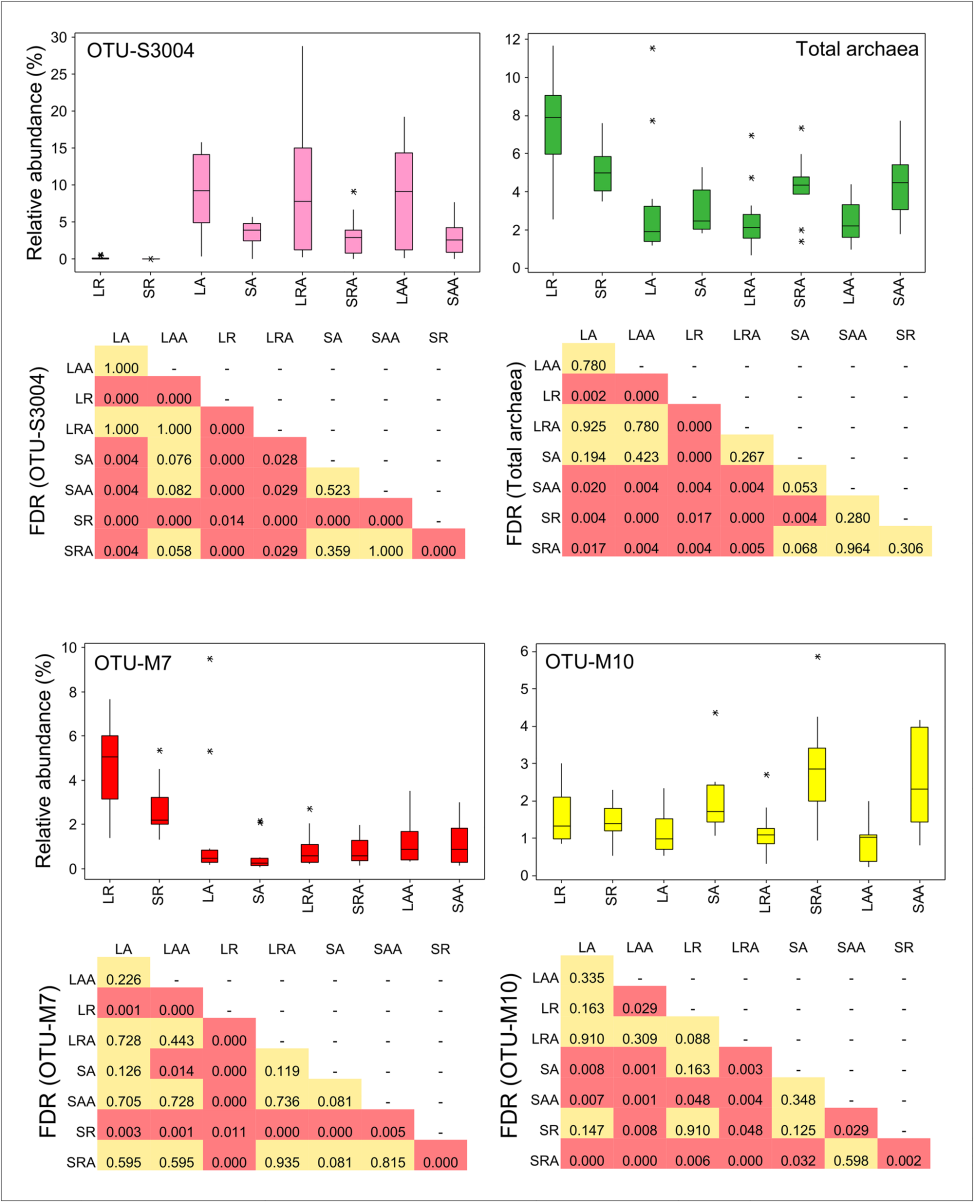
Relative abundances of OTUs S3004, M7 and M10 and total archaea. LR = Liquid Restricted (n = 14), SR = Solid Restricted (n = 14), LA = Liquid *Ad lib* (n = 13), SA = Solid *Ad lib* (n = 13), LRA = Liquid Restricted/*Ad lib* (n = 15), SRA = Solid Restricted/*Ad lib* (n = 15), LAA = Liquid *Ad lib*/*Ad lib* (n = 13), SAA = Solid *Ad lib*/*Ad lib* (n = 13). FDR = BH-corrected P values from Wilcoxon pairwise comparisons of relative abundances between different treatment groups.

By far the largest significant change (FDR<0.05) in both solid and liquid fractions was a decrease in group R, relative to the three *ad libitum* groups, of the relative abundance of OTU-S3004 which QIIME identified only as far as the family Succinivibrionaceae. Compared to group A, this single OTU accounted for an 8.7% (relative to all other OTUs) decrease in the group R liquid fraction and a 3.4% (relative to all other OTUs) decrease in the group R solid fraction. The relative abundance of OTU-S3004, while barely detectable in all group R animals, was detected as being high, particularly in the liquid fraction (30% in one of the liquid samples from group RA), in most of the animals in the *ad libitum* groups (Figs 4, 5 and 6). PCA showed that the rumen prokaryotic composition of *ad libitum* animals that had low levels of OTU-S3004 was more similar to group R (Fig 2) than group A. 96% of the 16S V4 sequences within this OTU were 100% identical suggesting that this is either mostly a single species or a group of very closely related bacteria. So it appears that there was an abundant population of an uncharacterised putative Succinivibrionaceae, mostly associated with the liquid fraction, that was dramatically reduced (88 fold in liquid and 425 fold in the solid fraction) by feed restriction in group R and then recovered to high levels upon return to an *ad libitum* diet in most of the group RA animals (Figs 4, 5 and 6). However, we do not know how many copies of the 16S rRNA gene are in the genome of this putative species so the relative abundance could be an overestimation of absolute abundance of the bacterial cells. The closest relatives to Succininvibrionaceae on the *rrn*DB 16S copy number data base [36] are members of the order Aeromonadales which have up to ten 16S rRNA gene copies per genome, although closely related species of bacteria can have different copy numbers of 16S genes in their genomes.

The most common members of the family Succinivibrionaceae reported so far in the bovine rumen are *Ruminobacter amylophilus*, *Succinivibrio dextrinosolvens*, and *Succinimonas amylolytica* [37]. A BLAST search of the OTU-S3004 consensus sequence against the NCBI nr/nt data base showed 100% identity with at least 96 uncultured rumen bacterium clone sequence accessions (e.g. accession numbers JF319383, HQ400400) [38, 39] and 3 uncultured bovine intestine bacteria accessions (accession numbers JX095261, JX095238, JX095110) [40] so this uncultured bacteria has probably been previously identified in the bovine rumen and gut.

QIIME assigned several other OTUs to *Succinivibrio* and *Ruminobacter* so it is unlikely that OTU-S3004 belongs to either of these genera. Interestingly, most of the other Succinivibrionaceae-assigned OTUs (including *Succinivibrio* and *Ruminobacter*) were more abundant in group R than in group A and so their response to feed restriction was opposite to that of OTU-S3004 (Fig 4, S1 Table).

Pope et al. (2011) proposed that the dominance of the Succinivibrionaceae species WG-1 in the foregut of macropods may be the reason for their low methane emissions [41]. Their proposal was based on the fact that hydrogen was previously shown to stimulate production of succinate as the principal fermentation end product in *Anaerobiospirillum succiniciproducens* which is a member of the Succinivibrionaceae [42], and that a number of other Succinivibrionaceae species also produce succinate [43]. Succinate is rapidly converted to propionate in the bovine rumen. So it is possible that Succinivibrioanceae compete with methanogens for hydrogen as a substrate for production of succinate/propionate rather than methane. However, although WG-1 was shown to produce succinate, they found that its production was not stimulated by hydrogen [41]. OTU-S3004 shares only 87% and 83% identity with the 16SrRNA V4 regions of WG-1 and *Anaerobiospirillum succiniciproducens* respectively so is unlikely to be closely related to either of these species.

Nevertheless there was a strong negative Spearman correlation (see later) between OTU-S3004 and the most abundant methanogenic archaea OTU in group R (*Methanobrevibacter*-assigned OTU-M7) which showed a reduction of similar magnitude in the opposite direction (i.e. high abundance (up to 8%) in group R and very low abundance in group A (apart from animals A2 and A17) (Fig 4). This suggests that OTU-S3004 may be competing with specific methanogen species for substrates such as hydrogen and the increased abundance of these methanogen species caused the dramatic reduction in abundance of OTU-S3004 in group R. It has been known for decades that increasing the intake of highly digestible diets in cattle leads to a decrease in methane production in the rumen [6, 44] but it is still not entirely clear why. It is possible that this unclassified putative Succinivibrionaceae species may be involved but this organism needs to be isolated and characterised to confirm this.

### Increased relative abundance of methanogens in feed restricted animals

Relative abundances of the sum of archaea-assigned OTUs were significantly higher (FDR<0.05) in both the liquid and solid fractions of group R compared to group A (Fig 6). This was mostly attributed to a significant increase in *Methanobrevibacter*-assigned OTU-M7 (Fig 6, S1 Table) which was significantly higher (FDR<0.05) in the group R liquid fraction than in the liquid and solid fractions of all the other groups (Fig 6). Compared to group A, OTU-M7 showed the largest increase out of all the OTUs in the group R liquid fraction (3.3% relative to all OTUs) and second largest increase of all the OTUs in the group R solid fraction (2.1% relative to all OTUs). This indicates that many of the methanogens were increased by feed restriction and decreased by the *ad libitum* diet. The reason for the increased relative abundance of methanogens in group R is probably due to reduced passage rate of feed through the rumen as a consequence of feed restriction and reduced daily intake. Growth rate of the methanogens tends to be negatively associated with passage rate of feed [45].

The dominant archaea OTUs, OTU-M7 and OTU-M10 (Fig 4), were both assigned to the genus *Methanobrevibacter* but, whereas the relative abundance of OTU-M7 was higher in group R than group A, the abundance of OTU-M10 was not different between these groups (Fig 6). As mentioned in the previous section, there was also a close negative relationship between relative abundance of OTU-S3004 and OTU-M7 but this was not evident for OTU-M10. A BLAST search against the NCBI 16S bacteria/archaea database showed that the closest match to a characterised species for OTU-M7 and OTU-M10 was *Methanobrevibacter millerae* (99% identity) and *Methanobrevibacter olleyae* (99% identity) respectively. Other close matches (S2 Table) to these OTUs show that OTU-M7 is probably from the *Methanobrevibacter gottschalkii* clade and OTU-M10 is from the *Methanobrevibacter ruminantium* clade [46]. Shi et al (2014) also found that relative abundances of organisms belonging to the *Methanobrevibacter gottschalkii* clade in sheep which were consistent high methane emitters [7]. *Methanobrevibacter millerae* (99% identity) and *Methanobrevibacter olleyae* can both utilise H_2_ + CO_2_ and formate + CO_2_ and do not differ significantly in any other growth requirements [47] so it is intriguing why OTU-M7 increased in group R whereas OTU-M10 did not. A recent report on phylogenetic marker analysis of the rumen microbiota of Sika deer fed different concentrations of tannin rich plants found that *Methanobrevibacter gottschalkii*, *Methanobrevibacter thaueri*, and *Methanobrevibacter millerae* responded differently than *Methanobrevibacter ruminantium* [48]. They suggested that this may because *Methanobrevibacter ruminantium* M1 is different to most other hydrogenotrophic methanogens as it lacks an *mcr*II (also called *mrt*) gene and contains only the *mcr*I system for the final methyl-CoM reduction step in methanogenesis [49]. The *mcr*II (*mrt*) gene codes for methyl CoM reductase II, an isoenzyme of methyl CoM reductase I, which is differentially regulated during growth to mediate methane formation at high partial pressures of hydrogen [50]. However, the draft genome sequences of *Methanobrevibacter millerae* strain DSM16643 and *Methanobrevibacter olleyae* strain DSM16632 on the JGI IMG/ER site both have *mcr*I and *mcr*II (*mrt*) genes. The lack of an *mcr*II gene in *Methanobrevibacter ruminantium* M1 might be strain specific. This will be apparent when genome sequences of other *Methanobrevibacter ruminantium* strains are completed. Nevertheless there are many other differences between the genomes of *Methanobrevibacter millerae* strain DSM16643 and *Methanobrevibacter olleyae* strain DSM16632. Including the number of protein coding genes (2383 for *M*. *millerae* and 1813 for *M*. *olleyae*), number of RNA genes (84 for *M*. *millerae* and 41 for *M*. *olleyae*) and number of tRNA genes (77 for *M*. *millerae* and 33 for *M*. *olleyae*). Now that gene sequence data is available for *M*. *millerae* and *M*. *olleyae*, it might be possible to determine why OTU-M7 and OTU-M10 respond differently in group R animals by deep metatranscriptomic analysis.

Relative abundance of OTUs assigned to the class Thermoplasmata was also increased in group R although abundance was less than 1% in all samples. However, it was recently reported that, although far lower in abundance than *Methanobrevibacter*, Thermoplasmata contribute significantly to methanogenesis in cattle rumen due to high levels of transcription [51]. These archaea are unusual in that they obtain energy and carbon from methylamines rather than H_2_/CO_2_ or H_2_/methanol, and they were recently reclassified as a new seventh order of methanogenic archaea called Methanomassiliicoccales [52].

### Relationship between putative Succinivibrionaceae and VFA

**Fig 7:**
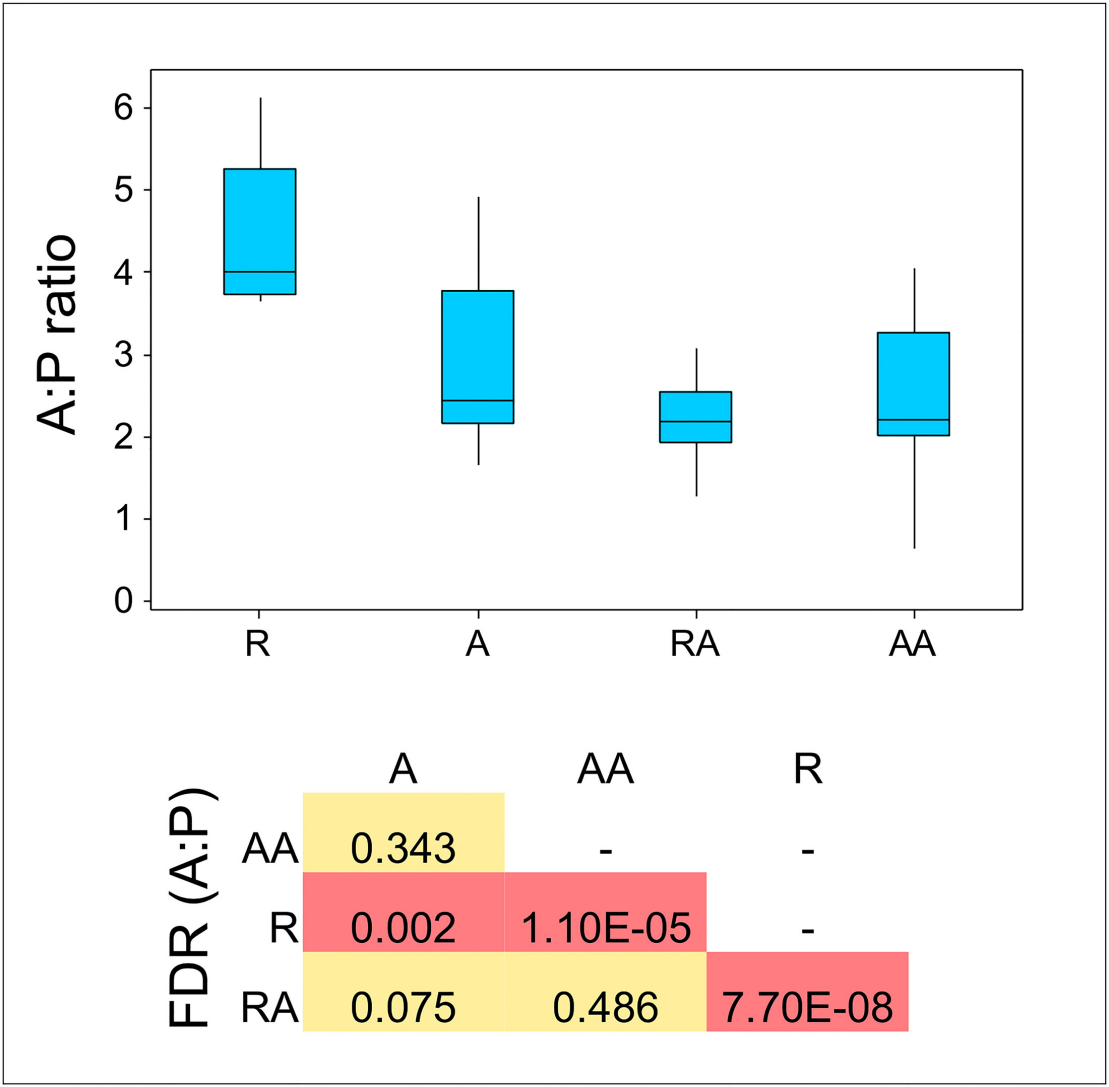
Acetate:Propionate (A:P) ratios in rumen samples from all groups. Group R (n = 14), A (n = 13), RA (n = 15) and AA (n = 13). Box and whisker plot showing median, quartiles, maximum and minimum values. FDR = BH-corrected P values derived from Wilcoxon pairwise comparisons of A:P ratios between different treatment groups.

Although we did not measure methane directly, we did measure rumen VFA concentrations and calculated the A:P ratio which is an indication of methanogenesis. The A:P ratio was significantly higher in the feed restricted group R than in the *ad libitum* groups A, RA and AA indicating increased methanogenesis in group R (Fig 7). Succinate does not accumulate in the rumen but is rapidly converted to propionate [53] so it is possible for succinate producing bacteria to influence the rumen A:P ratio. Compared to group A, in group R there was a 13.7% (P = 0.14) increase in absolute concentration of VFAs, a 26% increase in acetate (P = 0.054), 37% decrease in propionate (P = 0.04), a 68% increase in A:P (P = 1.86 x10^-5^) and a 104% increase in n-butyrate P = (0.0002). There were no significant differences in VFA concentrations between groups RA and AA. Even though their diet was the same, compared to group A, in group AA, there was a 48% (P = 0.0005) increase in absolute concentration of total VFAs, a 43% increase in acetate (P = 0.01), a 58% increase in propionate (P = 0.002), and a 44% increase in n-butyrate (P = 0.007) but no difference in A:P ratio. The increase in concentration of these VFAs in the older group AA bulls could be due to a variety of factors including increased overall VFA production, reduced VFA uptake, increased rumen size, or reduced VFA dilution due to lower water intake in the colder weather when the group AA samples were taken in November compared to September for the younger group A bulls. However, the fact that the A:P ratio was not different between groups A and AA reflects the lack of difference in relative abundance of rumen prokaryotes between these two groups.

**Fig 8:**
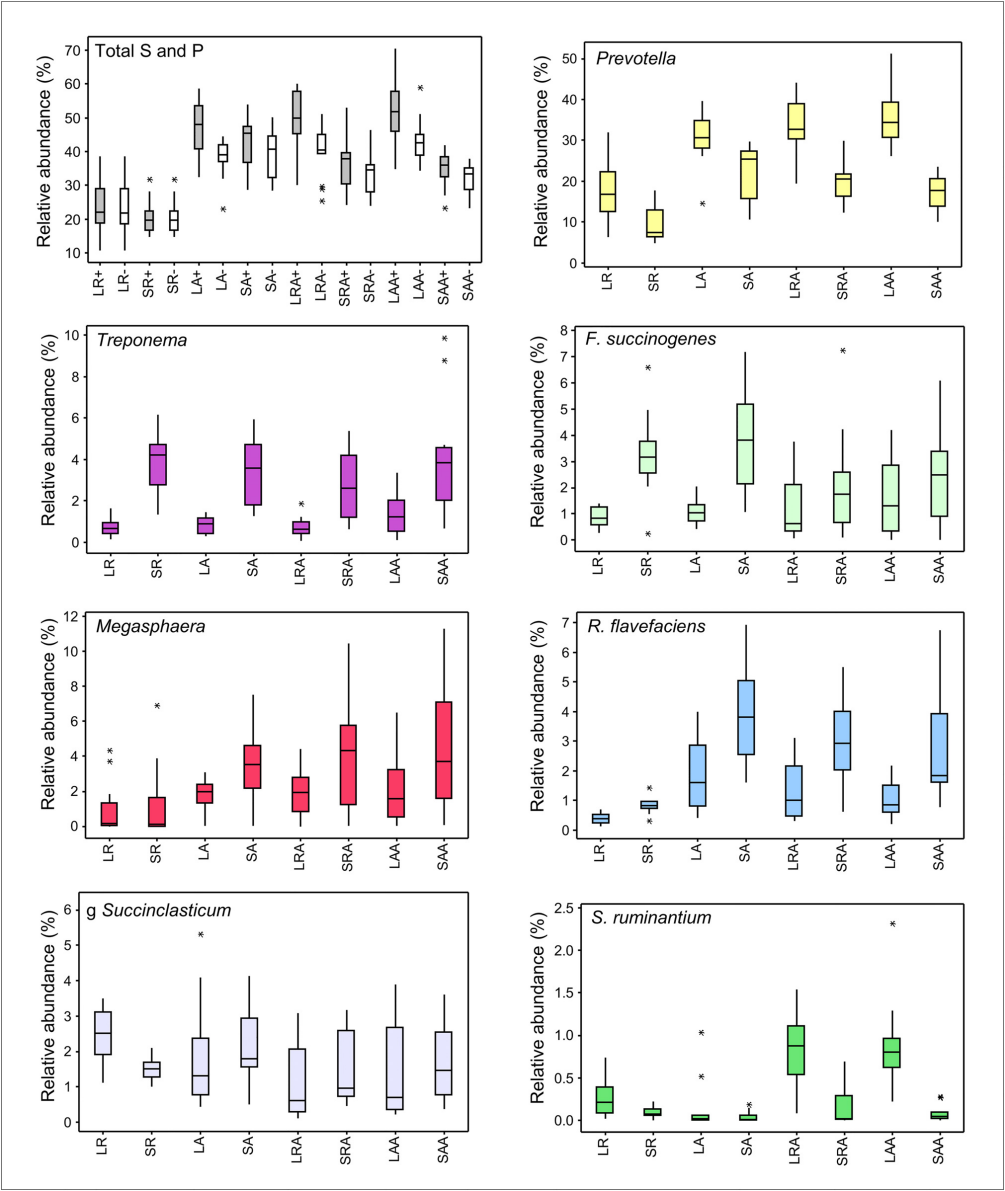
Differences in relative abundance of succinate and propionate producers between treatment groups. Box and whisker plots showing median, quartiles, maximum and minimum values, and outliers (asterisks) for the sum of relative abundances within each rumen sample of OTUs assigned to taxonomic groups (apart from Succinivibrionaceae) which are, or have members which are, succinate and propionate (S and P) producers. ‘Total S and P’ box and whisker plot shows sum of relative abundances of all OTUs assigned to S and P taxonomic groups excluding OTU-S3004 (white boxes-) and including OTU-S3004 (grey boxes +). LR = Liquid Restricted (n = 14), SR = Solid Restricted (n = 14), LA = Liquid *Ad lib* (n = 13), SA = Solid *Ad lib* (n = 13), LRA = Liquid Restricted/*Ad lib* (n = 15), SRA = Solid Restricted/*Ad lib* (n = 15), LAA = Liquid *Ad lib*/*Ad lib* (n = 13), SAA = Solid *Ad lib*/*Ad lib* (n = 13).

To try and determine the extent to which OTU-S3004 might influence rumen A:P ratio, we also looked at the abundance of OTUs which were assigned to 7 taxons (Veillonellaceae *Megasphaera*, *Ruminococcus flavefaciens*, *Prevotella*, *Selenomonas ruminantium*, Veillonellaceae *Succiniclasticum*, *Fibrobacter succinogenes*, Spirochaetaceae *Treponema*) that are, or contain members which are, known succinate and/or propionate (S and P) producers [54, 55] (Fig 8). The overall sum of the relative abundances of these S and P producers was reduced in group R, mainly due to decreases in *Prevotella*, *R*. *flavefaciens* and *Megasphaera*. Several of these taxonomic groups were clearly more associated with either the solid (e.g. *Treponema*, *R*. *flavefaciens*), or liquid (e.g. *Prevotella*) fraction. Addition of the relative abundance values for OTU-S3004 results in a large increase in overall abundance of S and P producers in the liquid fraction (Fig 8). *Selenomonas ruminantium*, which is thought to be one of the main rumen bacterial species that converts succinate to propionate and can account for up to 51% of the total viable bacterial count in the rumen [56, 57], but is undetectable under certain feeding regimes [58], was only detected as <1% in most animals in our study.

To look for possible relationships between VFA concentrations and OTU-S3004, archaea and known S and P producers, we performed Spearman’s rank correlation coefficient (*ρ*) analysis on liquid and solid fractions from combined treatment groups (Tables 1 and 2). This showed that for some of these taxonomic groups there were significant (P<0.05) correlations with propionate, n-butyrate and A:P ratio. The abundances of these taxonomic groups did not show significant Spearman correlations with acetate apart from a weak negative correlation with *R*. *flavefaciens* (solid fraction) and a positive correlation with Succinivibrionaceae (excluding OTU-S3004, liquid fraction). Only OTU-S3004 (liquid fraction only) and *Prevotella* (solid and liquid fractions) showed significant positive correlations with propionate plus significant negative correlations with A:P ratio. OTU-S3004 (liquid fraction) showed the most significant and strongest negative correlation with A:P ratio which further suggests that OTU-S3004 may be a producer of succinate and/or propionate in the rumen of cattle.

**Table 1:** Spearman correlation analysis between VFA and OTU-S3004, methanogenic archaea, and S and P producers in liquid rumen samples.

Taxonomic assignment	acetate		propionate		n-butyrate		A:P	
	*ρ*	P value	*ρ*	P value	*ρ*	P value	*ρ*	P value
*F*. *succinogenes*	+0.08	NS	+0.12	NS	-0.10	NS	-0.09	NS
*Treponema*	+0.22	NS	+0.15	NS	+0.15	NS	+0.09	NS
*Megasphaera*	-0.09	NS	-0.07	NS	-0.13	NS	0.00	NS
*Selenomonas*	-0.18	NS	+0.08	NS	-0.15	NS	-0.32	NS
*Succiniclasticum*	+0.03	NS	-0.30	<0.05	+0.33	<0.05	+0.46	<0.001
*R*. *flavefaciens*	-0.06	NS	+0.26	0.054	-0.43	<0.001	-0.36	<0.01
*Prevotella*	-0.01	NS	+0.51	<0.0001	0.00	NS	-0.57	<0.00001
Succinivibrionaceae excluding S3004	+0.41	<0.01	+0.15	NS	+0.51	<0.0001	+0.09	NS
Succinivibrionaceae including S3004	+0.05	NS	0.00	NS	-0.03	NS	-0.02	NS
OTU-S3004	-0.19	NS	+0.41	<0.01	-0.29	<0.05	-0.69	<1x10^-20^
OTU-M7	0.26	0.06	-0.37	<0.01	0.50	<0.0005	0.74	<1x10^-20^
OTU-M10	-0.05	NS	-0.32	<0.05	-0.16	NS	0.34	<0.05
Methanobacteria and Methanoicrobia	+0.21	NS	-0.45	<0.001	+0.34	<0.05	+0.75	<1x10^-20^
Thermoplasmata	+0.19	NS	-0.28	<0.05	+0.40	<0.01	+0.51	<0.0001

Values for relative abundances and VFA concentrations for all treatment groups (R, A, RA and AA) and rumen fractions (liquid and solid) were combined. *ρ* = Spearman’s rank correlation coefficient. NS = non-significant. A:P = acetate:propionate ratio.

**Table 2:** Spearman correlation analysis between VFA and OTU-S3004, methanogenic archaea, and S and P producers in solid rumen samples.

Taxonomic assignment	acetate		propionate		n-butyrate		A:P	
	*ρ*	P value	*ρ*	P value	*ρ*	P value	*ρ*	P value
*F*. *succinogenes*	-0.26	NS	-0.46	<0.001	-0.31	<0.05	+0.40	<0.01
*Treponema*	+0.07	NS	-0.21	NS	+0.08	NS	+0.39	<0.01
*Megasphaera*	0.00	NS	+0.17	NS	-0.18	NS	-0.29	<0.05
*Selenomonas*	+0.01	NS	+0.04	NS	+0.07	NS	-0.07	NS
*Succiniclasticum*	+0.09	NS	+0.01	NS	0.28	<0.05	+0.10	NS
*R*. *flavefaciens*	-0.28	<0.05	+0.19	NS	-0.58	<0.00001	-0.43	<0.001
*Prevotella*	-0.16	NS	+0.39	<0.01	-0.31	<0.05	-0.64	<0.000001
Succinivibrionaceae excluding S3004	-0.07	NS	0.00	NS	-0.23	NS	-0.13	NS
Succinivibrionaceae including S3004	-0.12	NS	+0.15	NS	-0.37	<0.01	-0.33	<0.05
OTU-S3004	-0.13	NS	+0.06	NS	-0.14	NS	-0.24	NS
OTU-M7	0.25	0.07	-0.29	<0.05	0.48	<0.001	0.69	<1x10^-20^
OTU-M10	-0.02	NS	0.34	<0.05	-0.05	NS	-0.31	<0.05
Methanobacteria and Methanomicrobia	0.13	NS	-0.07	NS	+0.39	<0.01	+0.35	<0.01
Thermoplasmata	-0.06	NS	-0.35	<0.01	+0.06	NS	+0.37	<0.01

Values for relative abundances and VFA concentrations for all treatment groups (R, A, RA and AA) and rumen fractions (liquid and solid) were combined. *ρ* = Spearman’s rank correlation coefficient. NS = non-significant. A:P = acetate:propionate ratio.

In the liquid fraction, archaea (Methanobacteria/Methanomicrobia and Thermoplasmata) showed highly significant positive correlations with A:P ratio and n-butyrate, and negative correlations with propionate, so it appears that the relationship between relative abundance of these archaea and VFAs was opposite to that of OTU-S3004. In both liquid and solid fractions, *Prevotella* and *R*. *flavefaciens* also showed correlations with propionate, butyrate and A:P that were opposite to those of archaea.

Out of all the Succinivibrionaceae and methanogen OTUs, the strongest negative and positive correlations with A:P were in the liquid fraction with OTU-S3004 and OTU-M7 respectively (S3 Table). In addition the Spearman correlation with A:P ratio was much stronger and more highly significant for OTU-M7 (*Methanobrevibacter gottschalkii* clade) than OTU-M10 (*Methanobrevibacter ruminantium* clade) (Tables 1 and 2). This supports the findings of Shi et al. (2014) who reported that the only archaea taxon that was increased in the rumens of sheep that were high methane emitters was the *Methanobrevibacter gottschalkii* clade [7].

### Correlation between Succinivibrionaceae and archaea

**Fig 9:**
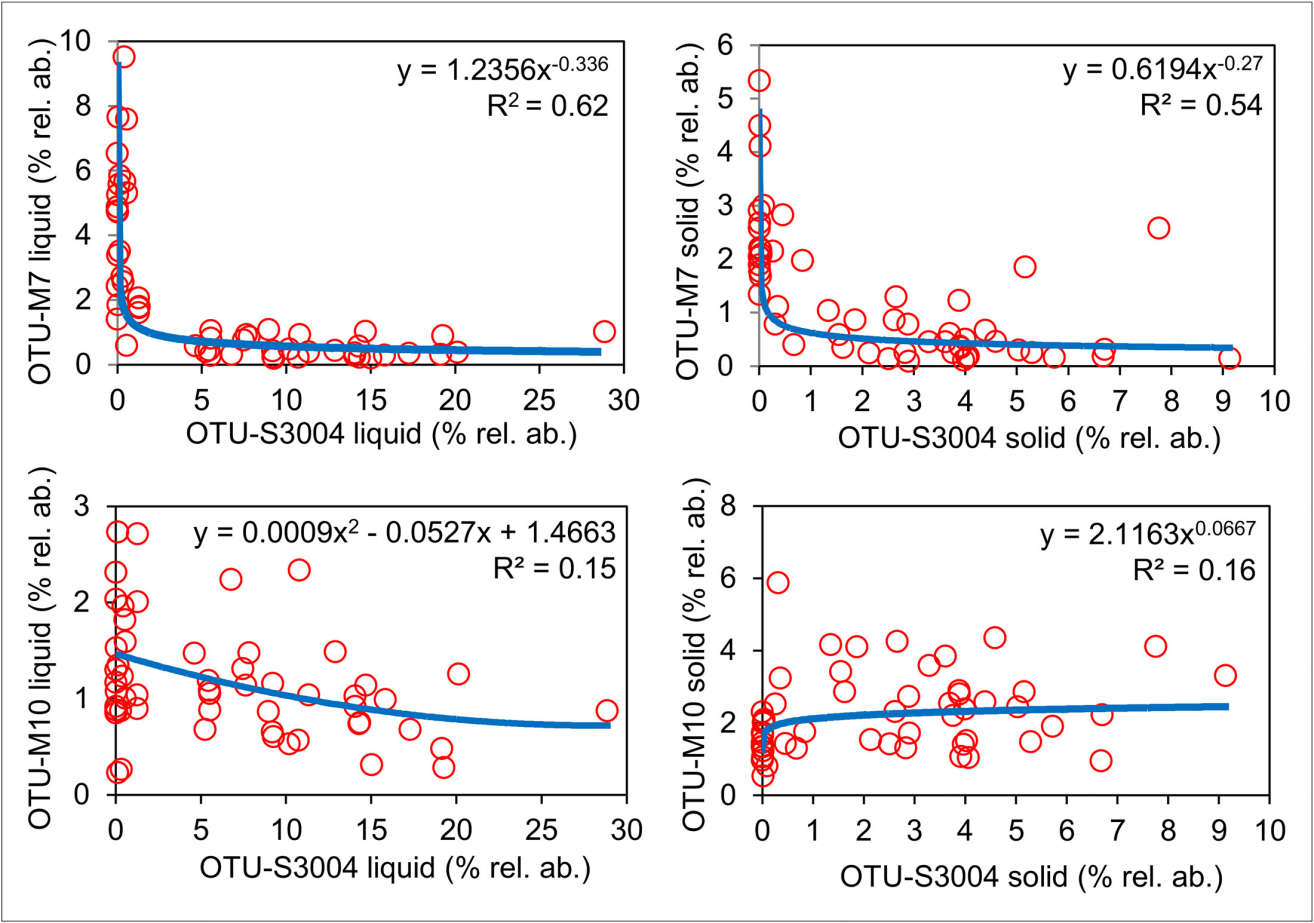
Correlation analysis of OTU-M7 and OTU-M10 with OTU-S3004. Scatter plot analysis of percentage relative abundances (% rel. ab.), in all liquid and solid fraction samples, of OTU-M7 (*Methanobrevibacter gottschalkii* clade) and OTU-M10 (*Methanobrevibacter ruminantium* clade) plotted against OTU-S3004. Power regression (blue dashed line) showed the highest R^2^ values (shown on each scatter plot) in all comparisons apart from OTU-M10 (liquid) vs. OTU-S3004 (liquid) for which a polynomial regression showed the highest R^2^ value.

To determine if OTU-S3004 might be specifically associated with OTU-M7, we looked at correlations between OTU-M7 and OTUs that showed the top 20 largest significant (FDR<0.05) changes in relative abundance between group R and group A (Tables 3 and 4). For this, the only negative correlation with OTU-M7 was with OTU-S3004 in the liquid fraction (*ρ* = -0.72, P value = <1x10^-20^). We also looked at correlations between the relative abundance of all of the Succinivibrionacae-assigned OTUs and all of the archaea-assigned OTUs (S4 Table). By far the strongest negative correlation in the liquid fraction was between OTU-M7 and OTU-S3004 (S4 Table). The only strong (i.e. *ρ* <-0.70) negative correlations in the solid fractions were between OTU-T26 and OTUs-S3008, S3010, and S3016 (S4 Table) which were low in relative abundance. *Prevotella* and *R*. *flavefaciens*-assigned OTUs also showed large decreases in group R (Fig 8) and significant negative Spearman correlations with M7 in both solid and liquid fractions (S5 and S6 Tables), but the relative abundance of these OTUs was low (<1%) and the level of significance and strength of correlation was far lower than for OTU-S3004. We also conducted scatter plot analysis to look at the relationship between the relative abundances of OTU-S3004 and the two dominant methanogen OTUs (OTUM7 and OTU-M10) (Fig 9). There was no obvious relationship between OTU-S3004 and OTU-M10 but there was a clear relationship between OTU-M7 and OTU-S3004 which was particularly strong for the liquid fraction samples and showed a power type regression. So there appeared to be a specific inverse relationship between the *Methanobrevibacter gottschalkii* clade, but not the *Methanobrevibacter ruminantium* clade, and OTU-S3004 in all of the liquid fraction samples. To our knowledge, a strong inverse relationship between this highly abundant putative *Succinivibrionaceae* and *Methanobrevibacter gottschalkii* clade in the rumen of cattle has not been previously reported and warrants further study.

**Table 3:** Spearman correlation analysis between OTU M7and OTUs showing the largest (top 20) changes in relative abundance between rumen liquid of group R and group A.

Taxon	OTU number	FDR	LR average	LA average	LR-LA	Spearman *ρ* with M7	Spearman P with M7
Succinivibrionaceae	3004	0.0005	0.100	8.755	-8.65	-0.72	<1X10^-20^
*Prevotella*	649	0.0009	0.092	4.930	-4.84	-0.24	NS
*Prevotella*	570	0.0005	0.029	2.882	-2.85	-0.10	NS
*Prevotella*	595	0.0006	0.053	2.270	-2.22	-0.17	NS
Clostridiales	1130	0.0005	0.056	1.760	-1.70	-0.12	NS
*Prevotella*	645	0.0014	0.066	1.709	-1.64	-0.01	NS
*Prevotella*	555	0.0005	0.016	1.566	-1.55	-0.01	NS
*Prevotella*	548	0.0005	0.013	1.456	-1.44	-0.08	NS
*Prevotella*	608	0.0005	0.010	1.195	-1.18	0.27	0.047
Lachnospiraceae	1823	0.00046	0.011	1.141	-1.13	-0.12	NS
*Bifidobacterium*	44	0.0074	1.165	0.014	1.15	0.27	0.048
*Butyrivibrio*	2052	0.0005	1.226	0.073	1.15	-0.21	NS
*Succiniclasticum*	2753	0.0050	2.155	0.718	1.44	-0.05	NS
*Ruminococcus*	2598	0.0046	1.910	0.449	1.46	-0.04	NS
Bacteroidales	203	0.0005	1.643	0.134	1.51	-0.28	NS
*Prevotella*	566	0.0011	2.382	0.667	1.71	0.19	NS
*Ruminococcus*	2625	0.0006	2.001	0.161	1.84	0.23	NS
*Prevotella*	571	0.0005	1.921	0.071	1.85	0.05	NS
*Butyrivibrio*	2050	0.0005	2.487	0.186	2.30	0.07	NS
*Methanobrevibacter*	7	0.0031	4.824	1.529	3.30	1.00	0.000

FDR = False discovery rate of change in relative abundance between group R and group A. LR average = average % relative abundance in the liquid fraction of group R. LA average = average % relative abundance in the liquid fraction of group A. LR-LA = LA average subtracted from LR average. Spearman *ρ* and Spearman P with M7 = Spearman’s rank correlation coefficient with relative abundance of OTU-M7 and corresponding P value. NS = non-significant (i.e. P > 0.05).

**Table 4:** Spearman correlation analysis between OTU M7and OTUs showing the largest (top 20) changes in relative abundance between rumen solid of group R and group A.

Taxon	OTU number	FDR	SR average	SA average	SR-SA	Spearman *ρ* with M7	Spearman P with M7
Succinivibrionaceae	3004	0.0004	0.008	3.438	-3.43	-0.24	0.08
*Prevotella*	570	0.0004	0.003	3.071	-3.07	-0.14	NS
*Prevotella*	608	0.0004	0.002	2.845	-2.84	-0.23	0.09
*Prevotella*	649	0.0014	0.022	2.380	-2.36	-0.08	NS
*Megasphaera*	2729	0.0155	1.088	3.315	-2.23	-0.12	NS
Clostridiales	1130	0.0009	0.048	2.126	-2.08	-0.04	NS
*Prevotella*	595	0.0007	0.020	1.684	-1.66	-0.16	NS
*Succiniclasticum*	2761	0.0010	0.001	1.551	-1.55	0.09	NS
*Prevotella*	645	0.0062	0.031	1.521	-1.49	0.12	NS
Lachnospiraceae	1831	0.0010	0.002	1.428	-1.43	-0.04	NS
*Butyrivibrio*	2075	0.0016	0.814	0.170	0.64	-0.05	NS
*Ruminococcus*	2598	0.0027	1.168	0.251	0.92	-0.08	NS
*Ruminococcus*	2594	0.0009	1.158	0.133	1.02	-0.08	NS
Clostridiales	1156	0.0031	1.361	0.322	1.04	0.09	NS
*Butyrivibrio*	2052	0.0004	1.203	0.091	1.11	-0.23	0.09
*Bifidobacterium*	44	0.0007	1.212	0.028	1.18	0.09	NS
*Ruminococcus*	2625	0.0006	1.612	0.103	1.51	0.00	NS
Bacteroidales	203	0.0009	1.997	0.230	1.77	0.02	NS
*Methanobrevibacter*	7	0.0011	2.686	0.545	2.14	1.00	0.000
*Butyrivibrio*	2050	0.0006	2.424	0.233	2.19	0.01	NS

FDR = False discovery rate of change in relative abundance between group R and group A. SR average = average % relative abundance in the solid fraction of group R. SA average = average % relative abundance in the solid fraction of group A. SR-SA = SA average subtracted from SR average. Spearman *ρ* and Spearman P with M7 = Spearman’s rank correlation coefficient with relative abundance of OTU-M7 and corresponding P value. NS = non-significant (i.e. P > 0.05).

## Conclusions

Phylogenetic amplicon sequencing showed that (i) the rumen prokaryotic communities of compensating animals at 55 days post-restriction were not significantly different from those of non-compensating animals (ii) the largest changes in relative abundance of rumen prokaryotes in feed restricted cattle compared with *ad libitum* cattle were an increase in the liquid fraction of the *Methanobrevibacter gottschalkii* clade and a strongly correlated, corresponding decrease in the liquid fraction of an uncultured Succinivibrionaceae species (OTU-S3004). Acetate:propionate ratios, which were higher in feed restricted animals, showed strong, highly significant correlations with *M*. *gottschalkii* clade (positive correlation) and OTU-S3004 (negative correlation). Propionate, but not acetate, was significantly correlated with the *M*. *gottschalkii* clade (negative correlation) and OTU-S3004 (positive correlation). Uncharacterised OTU-S3004 may have been a major contributor to the lower acetate:propionate ratios in *ad libitum* animals and showed a strong inverse relationship with the *M*. *gottschalkii* clade and therefore requires further study.

## Supporting Information

S1 Table Prokaryotic relative abundance table for all treatment groups. Relative abundances (%) of prokaryotic OTUs (comprised of sequence reads that share 97% sequence identity) and their taxonomic assignments in rumen solid and liquid fractions in groups R, A, RA and AA. Relative OTU abundances for individual animals are shown and P values, FDR are shown for comparison (T-test assuming unequal variance) between Log2 transformed relative abundance (%) for OTUs in groups LR and LA, SR and SA, LRA and LAA, SRA and SAA.
(XLSX)

S2 Table Results of NCBI BLAST search of OTU-M7 and OTU-M10 against the NCBI 16S bacteria/archaea database. (XLSX)

S3 Table Spearman correlation analysis between VFA concentrations and relative abundances of individual archaea and Succinivibrionaceae-assigned OTUs. Methanobacteria/Microbia OTUs = M1-M20, Thermoplasmata OTUs = T21-T27 and Succinivibrionaceae OTUs = S3000-S3016. P values shown as 0 are <1x10^-20^.
(PDF)

S4 Table Spearman correlation analysis between relative abundances of individual archaea and Succinivibrionaceae-assigned OTUs. Methanobacteria/Microbia OTUs = M1-M20, Thermoplasmata OTUs = T21-T27 and Succinivibrionaceae OTUs = S3000-S3016. P values shown as 0 are <1x10^-20^.
(PDF)

S5 Table Spearman correlation analysis between relative abundances of individual archaea-assigned OTUs and *Prevotella*-assigned OTUs in the liquid fraction. Methanobacteria/Microbia OTUs = M1-M20, Thermoplasmata OTUs = T21-T27 and *Prevotella* OTUs = P499-P825. P values shown as 0 are <1x10^-20^.
(XLSX)

S6 Table Spearman correlation analysis between relative abundances of individual archaea-assigned OTUs and *R*. *flavefaciens*-assigned OTUs in the liquid fraction. Methanobacteria/Microbia OTUs = M1-M20, Thermoplasmata OTUs = T21-T27 and *R*. *flavefaciens* OTUs = F2660-F2685.
(XLSX)
