# Supplementary material for: Illumina MiSeq Phylogenetic Amplicon Sequencing Shows a Large Reduction of an Uncharacterised Succinivibrionaceae and an Increase of the Methanobrevibacter gottschalkii Clade in Feed Restricted Cattle
Source: PLoS One. 2015 Jul 30;10(7):e0133234. doi: 10.1371/journal.pone.0133234 (PMC4520551; doi:10.1371/journal.pone.0133234)
Supplement: S3 Table — Methanobacteria/Microbia OTUs = M1-M20, Thermoplasmata OTUs = T21-T27 and Succinivibrionaceae OTUs = S3000-S3016. P values shown as 0 are <1x10^-20^. (PDF) [file pone.0133234.s003.pdf]

	Liquid Spearman rho values				Liquid Spearman P values			
	acet	prop	n-but	A:P	acet	prop	n-but	A:P
M1	0.11	-0.37	0.22	0.50	0.431	0.005	0.102	9E-05
M2	-0.01	-0.02	-0.09	-0.08	0.914	0.866	0.523	0.578
M3	-0.06	-0.30	0.05	0.23	0.678	0.026	0.714	0.086
M4	-0.16	0.03	-0.16	-0.20	0.244	0.828	0.242	0.139
M5	0.21	0.06	0.14	0.12	0.118	0.654	0.322	0.38
M6	0.08	-0.07	0.24	0.17	0.561	0.635	0.072	0.213
M7	0.26	-0.37	0.50	0.74	0.06	0.005	9E-05	0
M8	0.09	-0.12	0.17	0.21	0.527	0.378	0.207	0.128
M9	-0.04	-0.44	0.18	0.47	0.793	7E-04	0.196	3E-04
M10	-0.05	-0.32	-0.16	0.34	0.71	0.018	0.249	0.011
M11	0.18	0.09	0.11	-0.01	0.2	0.498	0.439	0.923
M12	-0.02	-0.06	0.05	0.02	0.881	0.687	0.721	0.903
M13	-0.19	0.10	-0.36	-0.32	0.167	0.446	0.007	0.017
M14	0.24	0.23	0.14	-0.08	0.074	0.094	0.298	0.567
M15	0.21	0.14	0.20	-0.04	0.118	0.32	0.142	0.797
M16	0.12	-0.05	0.21	0.20	0.386	0.714	0.122	0.142
M17	-0.11	-0.59	0.14	0.59	0.44	2E-06	0.316	2E-06
M18	-0.31	-0.10	-0.41	-0.16	0.022	0.472	0.002	0.25
M19	-0.27	0.14	-0.43	-0.46	0.045	0.294	9E-04	5E-04
M20	-0.06	0.14	0.12	-0.16	0.689	0.291	0.363	0.23
T21	0.24	0.37	0.19	-0.27	0.083	0.005	0.16	0.05
T22	0.00	0.04	0.04	0.04	0.996	0.774	0.754	0.758
T23	-0.01	-0.26	0.02	0.31	0.927	0.053	0.877	0.021
T24	-0.33	-0.10	-0.21	-0.08	0.015	0.455	0.129	0.545
T25	-0.32	0.04	-0.37	-0.27	0.016	0.766	0.006	0.042
T26	-0.30	0.21	-0.51	-0.51	0.025	0.133	6E-05	6E-05
T27	0.38	-0.01	0.44	0.28	0.004	0.94	9E-04	0.035
S3000	0.34	-0.09	0.46	0.39	0.012	0.52	4E-04	0.004
S3001	0.02	-0.08	0.24	0.04	0.908	0.575	0.079	0.792
S3002	-0.04	0.42	-0.13	-0.59	0.764	0.001	0.326	2E-06
S3003	0.04	-0.55	0.30	0.66	0.787	1E-05	0.026	5E-08
S3004	-0.19	0.41	-0.29	-0.69	0.175	0.002	0.034	0
S3005	0.32	-0.16	0.39	0.42	0.018	0.251	0.003	0.002
S3006	0.32	-0.22	0.54	0.54	0.019	0.102	2E-05	2E-05
S3007	-0.08	0.32	-0.25	-0.45	0.581	0.016	0.068	6E-04
S3008	0.25	-0.29	0.44	0.52	0.065	0.031	8E-04	5E-05
S3009	0.31	-0.19	0.51	0.50	0.022	0.164	8E-05	1E-04
S3010	0.07	-0.43	0.22	0.50	0.591	9E-04	0.11	1E-04
S3011	-0.01	-0.45	0.12	0.55	0.961	6E-04	0.366	1E-05
S3012	0.07	0.39	0.01	-0.47	0.63	0.004	0.967	3E-04
S3013	0.03	-0.41	0.17	0.48	0.854	0.002	0.222	2E-04
S3014	0.16	0.12	0.29	-0.05	0.244	0.365	0.029	0.739
S3015	-0.34	0.20	-0.43	-0.48	0.012	0.138	9E-04	2E-04
S3016	0.23	-0.43	0.40	0.68	0.098	0.001	0.002	1E-08

	Solid Spearman rho values				Solid Spearman P values			
	acet	prop	n-but	A:P	acet	prop	n-but	A:P
M1	-0.12	-0.60	0.12	0.64	0.386	1E-06	0.396	1E-07
M2	-0.11	0.22	-0.03	-0.29	0.432	0.104	0.814	0.033
M3	-0.06	-0.24	0.02	0.19	0.638	0.079	0.864	0.162
M4	-0.17	-0.01	-0.26	-0.18	0.207	0.97	0.051	0.182
M5	0.17	0.01	0.43	0.15	0.225	0.938	0.001	0.273
M6	-0.05	-0.36	0.04	0.45	0.711	0.007	0.749	7E-04
M7	0.25	-0.29	0.48	0.69	0.07	0.029	2E-04	0
M8	0.14	-0.03	0.32	0.24	0.299	0.805	0.017	0.072
M9	0.33	0.13	0.35	0.13	0.014	0.36	0.01	0.34
M10	-0.02	0.34	-0.05	-0.31	0.874	0.012	0.744	0.021
M11	-0.08	-0.17	0.17	0.12	0.584	0.213	0.21	0.364
M12	0.13	-0.19	0.26	0.35	0.326	0.164	0.051	0.01
M13	0.24	0.21	0.37	-0.04	0.083	0.119	0.006	0.795
M14	0.15	0.21	0.05	-0.14	0.271	0.121	0.7	0.297
M15	0.06	0.33	0.09	-0.33	0.687	0.013	0.5	0.015
M16	0.16	0.12	0.21	0.04	0.238	0.368	0.119	0.782
M17	0.00	-0.59	0.19	0.69	0.997	3E-06	0.16	1E-08
M18	0.03	0.10	-0.10	-0.15	0.845	0.464	0.49	0.274
M19	-0.23	-0.04	-0.22	-0.21	0.091	0.775	0.113	0.127
M20	0.22	0.03	0.41	0.20	0.104	0.841	0.002	0.145
T21	0.17	0.00	0.28	0.14	0.208	0.984	0.037	0.312
T22	-0.07	-0.30	0.18	0.34	0.601	0.024	0.193	0.012
T23	-0.03	-0.29	0.04	0.35	0.809	0.029	0.789	0.01
T24	-0.41	-0.16	-0.19	-0.10	0.002	0.231	0.159	0.475
T25	-0.19	-0.01	-0.17	-0.26	0.17	0.924	0.215	0.058
T26	-0.19	0.43	-0.43	-0.59	0.163	0.001	1E-03	2E-06
T27	0.07	-0.29	0.17	0.43	0.621	0.034	0.215	0.001
S3000	0.19	-0.24	0.37	0.45	0.175	0.073	0.005	6E-04
S3001	0.30	0.02	0.43	0.29	0.028	0.884	1E-03	0.033
S3002	-0.07	0.32	-0.10	-0.45	0.614	0.019	0.451	5E-04
S3003	0.04	-0.60	0.23	0.73	0.784	1E-06	0.096	0
S3004	-0.13	0.06	-0.14	-0.24	0.326	0.645	0.295	0.076
S3005	0.29	-0.13	0.50	0.42	0.034	0.349	9E-05	0.001
S3006	0.38	-0.16	0.56	0.46	0.004	0.245	1E-05	5E-04
S3007	-0.26	0.25	-0.34	-0.49	0.054	0.07	0.012	2E-04
S3008	0.29	-0.28	0.45	0.52	0.034	0.035	6E-04	5E-05
S3009	0.24	-0.30	0.40	0.50	0.073	0.028	0.002	1E-04
S3010	0.18	-0.41	0.42	0.61	0.185	0.002	0.001	7E-07
S3011	0.21	-0.14	0.35	0.37	0.126	0.316	0.009	0.005
S3012	-0.14	0.28	-0.23	-0.49	0.302	0.035	0.089	2E-04
S3013	0.21	-0.24	0.43	0.50	0.127	0.072	0.001	9E-05
S3014	0.18	0.06	0.38	0.17	0.183	0.638	0.005	0.211
S3015	-0.19	0.40	-0.36	-0.58	0.167	0.002	0.008	4E-06
S3016	0.28	-0.37	0.43	0.61	0.042	0.006	1E-03	7E-07
