# Supplementary material for: Illumina MiSeq Phylogenetic Amplicon Sequencing Shows a Large Reduction of an Uncharacterised Succinivibrionaceae and an Increase of the Methanobrevibacter gottschalkii Clade in Feed Restricted Cattle
Source: PLoS One. 2015 Jul 30;10(7):e0133234. doi: 10.1371/journal.pone.0133234 (PMC4520551; doi:10.1371/journal.pone.0133234)
Supplement: S4 Table — Methanobacteria/Microbia OTUs = M1-M20, Thermoplasmata OTUs = T21-T27 and Succinivibrionaceae OTUs = S3000-S3016. P values shown as 0 are <1x10^-20^. (PDF) [file pone.0133234.s004.pdf]

Liquid Spearman rho values

	M1	M2	M3	M4	M5	M6	M7	M8	M9	M10	M11	M12	M13	M14	M15	M16	M17	M18	M19	M20	T21	T22	T23	T24	T25	T26	T27
S3000	0.32	-0.11	0.36	-0.06	-0.08	0.15	0.55	0.28	0.42	0.08	0.24	0.19	-0.24	-0.16	0.10	0.40	0.46	-0.18	-0.43	-0.37	-0.10	0.22	0.09	-0.16	-0.28	-0.52	0.46
S3001	0.02	0.08	0.27	-0.10	-0.17	0.11	0.17	0.06	0.10	0.07	0.02	0.04	-0.18	-0.04	0.02	0.15	0.12	0.01	-0.15	0.01	0.10	-0.13	-0.09	-0.02	0.23	-0.33	0.20
S3002	-0.25	0.24	-0.16	0.49	0.20	0.23	-0.50	0.05	-0.33	-0.16	0.27	0.23	0.26	0.26	0.17	-0.29	-0.37	0.24	0.53	0.24	0.30	-0.14	-0.23	0.08	0.14	0.43	-0.15
S3003	0.64	-0.17	0.18	-0.18	-0.05	0.15	0.66	0.27	0.67	0.26	0.18	0.27	-0.24	-0.16	0.12	0.30	0.80	-0.06	-0.35	-0.21	-0.41	0.04	0.17	-0.11	-0.22	-0.49	0.48
S3004	-0.25	0.20	-0.22	0.26	-0.09	-0.06	-0.72	-0.21	-0.40	-0.35	0.09	0.08	0.25	0.06	0.25	-0.27	-0.36	0.22	0.51	0.12	0.12	-0.13	-0.12	0.10	0.32	0.51	-0.29
S3005	0.15	0.01	0.17	-0.13	0.15	0.05	0.57	0.17	0.34	0.30	-0.06	-0.06	-0.18	0.17	-0.18	0.08	0.24	-0.01	-0.40	-0.05	-0.03	0.00	0.04	-0.16	-0.30	-0.48	0.43
S3006	0.29	-0.13	0.26	-0.01	0.02	0.22	0.66	0.27	0.62	0.05	0.08	0.09	-0.29	-0.20	0.11	0.41	0.50	-0.25	-0.49	-0.22	0.01	0.12	0.13	0.02	-0.43	-0.55	0.37
S3007	-0.33	0.29	-0.21	0.39	-0.15	-0.20	-0.53	-0.14	-0.40	-0.13	0.09	0.04	0.36	0.09	0.18	-0.11	-0.53	0.19	0.63	-0.02	0.01	-0.01	-0.12	-0.01	0.15	0.47	-0.29
S3008	0.38	-0.21	0.24	-0.18	0.08	0.02	0.66	0.27	0.50	0.18	0.08	0.09	-0.20	-0.24	0.09	0.37	0.61	-0.18	-0.46	-0.13	-0.15	0.18	0.11	-0.02	-0.38	-0.61	0.51
S3009	0.21	0.03	0.36	-0.11	0.08	0.20	0.63	0.32	0.53	-0.01	0.15	0.09	-0.26	-0.18	0.06	0.37	0.46	-0.24	-0.48	-0.16	0.01	0.14	0.01	-0.01	-0.39	-0.59	0.31
S3010	0.22	0.03	0.19	-0.03	0.15	0.04	0.51	0.28	0.39	0.26	-0.03	0.05	-0.19	-0.17	-0.05	0.10	0.39	-0.13	-0.39	-0.15	-0.11	0.04	0.13	0.12	-0.50	-0.38	0.35
S3011	0.25	-0.18	0.25	-0.13	-0.01	-0.02	0.44	0.13	0.34	0.14	-0.14	-0.11	-0.18	-0.12	-0.16	0.09	0.43	-0.05	-0.47	-0.05	-0.20	0.15	0.07	0.14	-0.24	-0.37	0.25
S3012	-0.23	0.14	0.03	0.00	-0.40	-0.26	-0.36	-0.16	-0.17	-0.19	0.24	0.15	0.07	0.12	0.05	0.05	-0.23	0.12	0.18	-0.02	-0.02	0.16	-0.15	-0.04	0.18	0.27	-0.09
S3013	0.28	-0.24	0.31	-0.05	0.12	0.10	0.51	0.21	0.49	0.21	0.07	0.16	-0.18	-0.27	-0.01	0.15	0.54	-0.07	-0.46	-0.08	-0.13	0.13	0.14	0.03	-0.30	-0.50	0.23
S3014	0.03	-0.12	-0.01	0.01	-0.32	-0.07	0.05	-0.06	0.03	0.05	0.03	0.02	-0.27	-0.06	-0.06	0.05	0.08	-0.33	-0.26	-0.18	0.03	0.00	-0.06	0.11	-0.10	-0.18	0.22
S3015	-0.33	0.34	-0.11	0.32	-0.09	0.05	-0.58	-0.08	-0.45	-0.17	0.02	0.09	0.33	0.38	0.13	-0.34	-0.44	0.26	0.52	0.06	0.27	0.00	-0.26	0.20	0.26	0.70	-0.34
S3016	0.51	-0.07	0.11	-0.18	0.11	0.18	0.69	0.25	0.48	0.26	0.08	0.10	-0.34	0.03	-0.01	0.24	0.58	-0.18	-0.50	-0.01	-0.13	0.06	0.28	0.06	-0.49	-0.51	0.48

Liquid Spearman P values

	M1	M2	M3	M4	M5	M6	M7	M8	M9	M10	M11	M12	M13	M14	M15	M16	M17	M18	M19	M20	T21	T22	T23	T24	T25	T26	T27
S3000	0.02	0.43	0.01	0.66	0.58	0.29	0	0.04	0	0.57	0.08	0.16	0.08	0.24	0.45	0	0	0.2	0	0.01	0.45	0.1	0.5	0.25	0.04	0	0
S3001	0.91	0.58	0.05	0.48	0.22	0.41	0.22	0.68	0.47	0.63	0.87	0.78	0.18	0.77	0.89	0.27	0.37	0.93	0.27	0.93	0.47	0.36	0.52	0.86	0.09	0.01	0.15
S3002	0.07	0.07	0.25	0	0.15	0.1	0	0.74	0.02	0.23	0.05	0.09	0.06	0.06	0.21	0.03	0.01	0.08	0	0.07	0.03	0.3	0.1	0.54	0.31	0	0.27
S3003	0	0.23	0.19	0.19	0.69	0.28	0	0.05	0	0.06	0.19	0.05	0.08	0.24	0.4	0.03	0	0.67	0.01	0.13	0	0.77	0.21	0.42	0.11	0	0
S3004	0.06	0.13	0.1	0.05	0.54	0.65	0	0.12	0	0.01	0.52	0.58	0.06	0.65	0.07	0.05	0.01	0.11	0	0.36	0.39	0.34	0.37	0.48	0.02	0	0.03
S3005	0.26	0.93	0.22	0.34	0.29	0.71	0	0.22	0.01	0.02	0.69	0.65	0.18	0.21	0.19	0.54	0.07	0.94	0	0.7	0.81	0.98	0.78	0.23	0.03	0	0
S3006	0.03	0.33	0.05	0.93	0.9	0.1	0	0.04	0	0.72	0.56	0.52	0.03	0.15	0.43	0	0	0.07	0	0.11	0.95	0.39	0.36	0.88	0	0	0.01
S3007	0.01	0.03	0.12	0	0.27	0.15	0	0.3	0	0.36	0.53	0.8	0.01	0.52	0.19	0.44	0	0.17	0	0.91	0.94	0.96	0.37	0.92	0.27	0	0.03
S3008	0	0.12	0.08	0.2	0.55	0.87	0	0.04	0	0.19	0.55	0.49	0.15	0.08	0.52	0.01	0	0.19	0	0.35	0.27	0.18	0.43	0.91	0	0	0
S3009	0.12	0.84	0.01	0.41	0.58	0.14	0	0.02	0	0.96	0.27	0.5	0.06	0.2	0.66	0.01	0	0.08	0	0.23	0.93	0.29	0.93	0.93	0	0	0.02
S3010	0.11	0.85	0.17	0.85	0.28	0.75	0	0.04	0	0.06	0.84	0.71	0.16	0.23	0.73	0.45	0	0.34	0	0.28	0.44	0.78	0.34	0.39	0	0	0.01
S3011	0.06	0.18	0.06	0.35	0.97	0.89	0	0.33	0.01	0.3	0.3	0.41	0.2	0.37	0.24	0.52	0	0.73	0	0.71	0.15	0.28	0.62	0.3	0.08	0.01	0.07
S3012	0.09	0.29	0.83	0.98	0	0.06	0.01	0.25	0.21	0.16	0.08	0.28	0.62	0.38	0.72	0.73	0.08	0.39	0.18	0.86	0.87	0.23	0.26	0.76	0.19	0.04	0.5
S3013	0.04	0.07	0.02	0.71	0.36	0.45	0	0.13	0	0.13	0.63	0.24	0.2	0.05	0.92	0.29	0	0.63	0	0.57	0.34	0.35	0.3	0.82	0.03	0	0.1
S3014	0.84	0.37	0.95	0.92	0.02	0.64	0.71	0.65	0.81	0.74	0.83	0.87	0.05	0.67	0.66	0.7	0.58	0.01	0.06	0.18	0.83	0.98	0.64	0.44	0.47	0.19	0.1
S3015	0.01	0.01	0.45	0.02	0.52	0.74	0	0.58	0	0.2	0.88	0.52	0.01	0	0.35	0.01	0	0.06	0	0.66	0.04	0.99	0.05	0.13	0.06	0	0.01
S3016	0	0.6	0.42	0.19	0.41	0.19	0	0.06	0	0.06	0.57	0.45	0.01	0.83	0.94	0.08	0	0.19	0	0.93	0.34	0.66	0.04	0.66	0	0	0

Solid Spearman rho values

	M1	M2	M3	M4	M5	M6	M7	M8	M9	M10	M11	M12	M13	M14	M15	M16	M17	M18	M19	M20	T21	T22	T23	T24	T25	T26	T27
S3000	0.39	-0.24	0.1	-0.14	0.26	0.37	0.48	0.14	0.05	-0.24	0.27	0.15	0.06	-0.13	-0.25	0.14	0.39	-0.05	-0.21	0.17	0.15	0.3	0.03	-0.01	-0.17	-0.52	0.3
S3001	0.22	-0.11	0.13	-0.2	0.26	0.23	0.4	0.28	0.25	-0.08	-0.03	0.36	-0.04	-0.08	0.03	0.03	0.26	-0.03	-0.27	0.05	0.2	0.07	0.19	-0.24	0.03	-0.33	0.26
S3002	-0.58	0.39	-0.27	-0.13	-0.11	-0.47	-0.32	-0.13	-0.07	0.34	-0	-0.06	-0.07	-0.05	0.17	-0.17	-0.58	-0.16	0.29	-0.2	-0.04	-0.32	-0.25	0.03	0.04	0.36	-0.27
S3003	0.78	-0.36	0.24	-0.05	0.19	0.47	0.6	0.24	0.04	-0.4	0.19	0.44	-0.01	-0.14	-0.35	0.1	0.9	-0.09	-0.23	0.22	0.02	0.44	0.37	0.01	-0.04	-0.65	0.43
S3004	-0.09	-0.01	0.18	0.06	-0.11	-0.34	-0.24	-0.08	-0.29	-0.05	-0.13	-0.02	0.15	0.05	-0.07	-0.21	-0.08	-0.04	0.18	0.13	-0.03	-0.12	0.04	-0.03	0.06	0.16	-0.32
S3005	0.44	-0.21	0.15	-0.07	0.41	0.27	0.55	0.36	0.24	-0.15	0.21	0.43	0.22	-0.1	0	0.18	0.5	0.09	-0.18	0.2	0.27	0.38	0.37	-0.04	-0.12	-0.52	0.31
S3006	0.26	-0.22	0.1	-0.12	0.37	0.25	0.52	0.23	0.28	-0.35	0.31	0.13	0.18	-0.16	-0.17	0.29	0.39	-0.17	-0.1	0.14	0.15	0.33	0.04	-0.08	-0.27	-0.62	0.32
S3007	-0.53	0.49	-0.06	0.16	-0.22	-0.31	-0.56	-0.27	-0.2	0.27	-0.06	-0.19	-0.06	0.17	0.16	-0.23	-0.6	0.01	0.33	-0.07	0.07	-0.2	-0.27	0.23	-0.06	0.59	-0.46
S3008	0.45	-0.33	0.29	0.06	0.23	0.26	0.53	0.29	0.11	-0.37	0.22	0.29	0.12	-0.21	-0.18	0.19	0.5	-0.04	-0.14	0.21	0.11	0.4	0.3	-0.11	-0.17	-0.71	0.4
S3009	0.42	-0.31	0.21	0.1	0.29	0.31	0.5	0.27	0.09	-0.36	0.3	0.29	0.17	-0.15	-0.3	0.19	0.46	-0.11	-0.11	0.29	0.09	0.39	0.23	-0.01	-0.19	-0.67	0.39
S3010	0.53	-0.28	0.42	-0.09	0.28	0.39	0.56	0.19	0.02	-0.37	0.35	0.39	0.16	-0.12	-0.23	0.06	0.6	0.07	-0.21	0.18	0.12	0.36	0.3	-0.06	-0.21	-0.74	0.43
S3011	0.37	-0.16	0.01	-0.06	0.24	0.16	0.53	0.17	0.09	-0.16	0.14	0.39	0.02	-0.04	-0.28	0.03	0.4	-0.02	-0.18	0.15	-0.02	0.24	0.2	-0.21	-0.15	-0.55	0.43
S3012	-0.44	0.06	-0.14	0.02	-0.18	-0.32	-0.42	-0.18	-0.13	0.27	0.05	0.02	0.03	0.11	0.06	-0.1	-0.41	0.29	0.17	-0.18	-0	-0.14	-0.3	0.07	-0.01	0.34	-0.31
S3013	0.46	-0.34	0.25	0.05	0.39	0.29	0.62	0.31	0.12	-0.33	0.2	0.28	0.17	-0.21	-0.12	0.18	0.52	-0.09	-0.21	0.24	0.2	0.33	0.3	-0.15	-0.21	-0.63	0.36
S3014	0.1	-0.09	-0.09	-0.06	0.3	0.13	0.44	0.18	0.33	0.07	0.25	0.05	0.12	0.06	-0.13	0.12	-0.03	-0	-0.27	0.39	0.06	0.2	-0.23	0.07	-0.37	-0.37	0.27
S3015	-0.6	0.23	-0.2	0.04	-0.33	-0.41	-0.59	-0.33	-0.12	0.32	-0.28	-0.32	0.01	-0.05	0.24	-0.02	-0.61	0.03	0.16	-0.06	0.01	-0.27	-0.28	0.03	-0.02	0.67	-0.55
S3016	0.54	-0.37	0.22	-0	0.33	0.35	0.62	0.4	0.07	-0.38	0.26	0.46	0.13	-0.18	-0.22	0.08	0.63	-0.03	-0.15	0.26	0.18	0.41	0.36	-0.16	-0.18	-0.73	0.4

Solid Spearman P values

	M1	M2	M3	M4	M5	M6	M7	M8	M9	M10	M11	M12	M13	M14	M15	M16	M17	M18	M19	M20	T21	T22	T23	T24	T25	T26	T27
S3000	0	0.07	0.46	0.29	0.06	0.01	0	0.3	0.7	0.08	0.04	0.27	0.69	0.34	0.07	0.29	0	0.71	0.13	0.2	0.27	0.03	0.81	0.94	0.21	0	0.03
S3001	0.11	0.42	0.34	0.14	0.06	0.09	0	0.04	0.07	0.57	0.84	0.01	0.78	0.57	0.84	0.8	0.06	0.84	0.05	0.72	0.14	0.59	0.16	0.07	0.84	0.01	0.05
S3002	0	0	0.05	0.36	0.41	0	0.02	0.34	0.6	0.01	0.98	0.65	0.63	0.69	0.21	0.21	0	0.23	0.03	0.14	0.77	0.02	0.06	0.85	0.75	0.01	0.05
S3003	0	0.01	0.08	0.72	0.17	0	0	0.07	0.77	0	0.17	0	0.93	0.29	0.01	0.45	0	0.52	0.09	0.11	0.9	0	0.01	0.93	0.77	0	0
S3004	0.51	0.97	0.18	0.65	0.42	0.01	0.08	0.58	0.03	0.73	0.35	0.9	0.26	0.7	0.6	0.13	0.57	0.76	0.19	0.34	0.81	0.39	0.78	0.81	0.68	0.23	0.02
S3005	0	0.12	0.26	0.61	0	0.04	0	0.01	0.07	0.26	0.12	0	0.11	0.47	0.98	0.18	0	0.53	0.19	0.14	0.04	0	0.01	0.76	0.36	0	0.02
S3006	0.05	0.1	0.46	0.39	0.01	0.06	0	0.1	0.04	0.01	0.02	0.35	0.19	0.25	0.22	0.03	0	0.22	0.47	0.31	0.28	0.02	0.76	0.55	0.05	0	0.02
S3007	0	0	0.68	0.24	0.11	0.02	0	0.05	0.15	0.05	0.65	0.17	0.68	0.22	0.26	0.1	0	0.93	0.01	0.61	0.62	0.14	0.05	0.09	0.68	0	0
S3008	0	0.01	0.03	0.67	0.08	0.06	0	0.03	0.42	0.01	0.11	0.03	0.39	0.12	0.2	0.16	0	0.76	0.32	0.13	0.43	0	0.02	0.41	0.22	0	0
S3009	0	0.02	0.12	0.48	0.03	0.02	0	0.04	0.5	0.01	0.02	0.03	0.22	0.27	0.03	0.15	0	0.41	0.42	0.03	0.51	0	0.09	0.95	0.17	0	0
S3010	0	0.04	0	0.53	0.04	0	0	0.16	0.9	0.01	0.01	0	0.26	0.38	0.09	0.64	0	0.63	0.13	0.19	0.37	0.01	0.03	0.68	0.12	0	0
S3011	0.01	0.24	0.92	0.64	0.08	0.25	0	0.2	0.53	0.24	0.3	0	0.9	0.78	0.04	0.85	0	0.9	0.19	0.28	0.88	0.08	0.15	0.12	0.28	0	0
S3012	0	0.69	0.32	0.89	0.18	0.02	0	0.19	0.36	0.05	0.73	0.89	0.83	0.41	0.65	0.45	0	0.03	0.23	0.18	0.99	0.31	0.03	0.63	0.93	0.01	0.02
S3013	0	0.01	0.07	0.72	0	0.03	0	0.02	0.37	0.01	0.15	0.04	0.22	0.12	0.38	0.19	0	0.52	0.13	0.08	0.13	0.01	0.02	0.28	0.12	0	0.01
S3014	0.48	0.54	0.5	0.67	0.03	0.36	0	0.18	0.01	0.63	0.07	0.73	0.36	0.68	0.36	0.39	0.8	0.98	0.05	0	0.65	0.15	0.1	0.61	0.01	0.01	0.05
S3015	0	0.09	0.14	0.77	0.01	0	0	0.01	0.39	0.02	0.04	0.02	0.91	0.74	0.07	0.91	0	0.85	0.25	0.66	0.93	0.05	0.04	0.83	0.91	0	0
S3016	0	0.01	0.11	0.98	0.01	0.01	0	0	0.62	0	0.06	0	0.34	0.2	0.11	0.57	0	0.82	0.29	0.06	0.19	0	0.01	0.23	0.19	0	0
